# The preference for potential in competence, not in morality: Asymmetric biases regarding a group's potential for moral improvement and decline

**DOI:** 10.1371/journal.pone.0236748

**Published:** 2020-08-04

**Authors:** Zhijie Xie, Fangfang Wen, Xiao Tan, Jin Wei, Bin Zuo

**Affiliations:** 1 School of Psychology, Central China Normal University, Wuhan, Hubei, China; 2 Center for Studies of Social Psychology, Central China Normal University, Wuhan, Hubei, China; 3 School of Psychology, Xinxiang Medical University, Xinxiang, Henan, China; Shandong University of Science and Technology, CHINA

## Abstract

The current study examined the effect of a group’s potential for improvement and decline in morality and competence on applicants’ willingness to join the group. We conducted four experiments with 399 Chinese participants who rated their willingness to join groups with potential for improvement, potential for decline, or stability in terms of morality or competence. The results showed that, compared with groups with stable competence, participants preferred groups with potential for competence improvement and were more averse to groups with potential for competence decline. However, the biases regarding the potential for moral improvement and decline were asymmetric. Specifically, compared with groups with stable morality, participants had no preference for groups with potential for moral improvement, but were more averse to groups with potential for moral decline. Possible explanations for the asymmetric biases regarding the potential for moral improvement and decline and future research directions are discussed.

## Introduction

Corporate morality plays an important role in attracting talent [1–5]. Imagine that an authoritative rating agency measured the corporate morality of almost all enterprises in an industry and published the results. You are an applicant who checked the morality rankings of three companies you have been interested in over the past five years, and found that one ranks moderately but has high potential for improvement, one ranks high but has high potential for decline, and one ranks between the other two companies and remains stable. Which company would you like the most? Which company would you like the least?

The answer to the first question may be consistent if we replace morality rankings with competence rankings—individuals may prefer the company with a high potential for improvement over the other two companies. Indeed, previous research showed a preference for individuals with high potential for improvement compared with individuals with high achievements [6, 7]. However, the preference for the potential for improvement was verified mostly in the field of competence, such as leadership [6–8], creativity [9], and academic ability [6]. Thus, it is unclear whether individuals prefer the potential for moral improvement over a high level of morality.

The second question has to do with the aversion to the potential for moral decline. Previous research showed a preference for individuals with a potential to reach high achievements relative to individuals who have reached those achievements [6]. However, it is unclear whether individuals with the potential to descend to mediocre performances are more disliked than those with mediocre performances.

### Symmetric biases regarding potential for competence improvement and decline

A productive starting point for exploring the biases in relation to potential in morality involves referring to research on potential in competence, as morality and competence are two fundamental dimensions in the judgment of individuals and groups [10–16]. Morality is linked to the perceived correctness of social targets, and is exemplified by traits such as honesty, sincerity, and trustworthiness [12, 16, 17], whereas competence is linked to goal-attainment and is exemplified by traits such as intelligence, skillfulness, and confidence [18–20]. In view of the predominant role of morality and competence in judging other individuals and groups, it is valuable to compare biases in relation to potential in the morality and competence dimensions.

Previous research on individuals’ preference for the potential for competence improvement [7, 8, 21] mostly adopted the paradigm of Experiment 3 in Tormala, Jia, & Norton’s study [6]. In that paradigm, participants were asked to evaluate an individual with high achievement and high potential for decline and an individual with moderate achievement and high potential for improvement. A more positive evaluation of the second individual suggested a preference for the potential for improvement. However, it is difficult to distinguish whether the more positive evaluation of the second individual was due to a preference for the potential for improvement or an aversion to the potential for decline.

Although a preference for the potential for individual competence improvement and an aversion to the potential for individual competence decline were mixed in previous studies, both these biases may exist when people process individual and group information. In some cases, a person’s processing of similar individual and group information will produce different results. For example, compared with groups, people judge the individuals more positively [22]. The negativity bias in honesty judgments and the positivity bias in intelligence judgments are more diminutive in trait judgments of groups than in trait judgments of individuals [23]. However, when facing a linear development trend of individuals, groups, and things, people tend to predict that the linear trend will continue into the future [24–26]. Therefore, when compared with high achievements with a horizontal trend, moderate achievements with the potential for improvement may lead to higher expected future achievements. In addition, individuals generally assign higher values to future achievements than to past achievements [27, 28]. Hence, there may be a preference for individuals and groups with a potential for improvement over those with stable high achievements. Similarly, compared with moderate achievements with a horizontal trend, high achievements with a potential for decline may lead to lower expected future achievements, resulting in an aversion to the potential for decline.

### Asymmetric biases regarding potential for moral improvement and decline

Individuals may prefer the potential for a group’s competence improvement and experience aversion to the potential for a group’s competence decline. However, at least two lines of research suggest that individuals may have a non-preference for the potential for a group’s moral improvement, but an aversion to the potential for a group’s moral decline. The first line includes experiments evaluating beliefs about others’ moral improvement and decline, which showed that people’s evaluations of moral and immoral behaviors are asymmetric [29–33]. For example, Klein and Epley [33] described a situation in which an accomplished professor who received an $80,000-grant for his research decided to donate $10,000 to a nonprofit institution dedicated to research on poverty; this action was rated as “nice” as donating the whole sum of $80,000. However, if this accomplished professor found a bag containing $80,000 and took all $80,000 for himself, this action was rated as less “nice” than taking $10,000 for himself and returning the rest to the police. Because moral behaviors are not mandatory, people’s evaluations of the growing good deeds will not increase in proportion. However, owing to low tolerance of immoral behaviors, sanctions against increasing immoral behaviors will become more and more prominent [29]. Therefore, these asymmetric evaluations of moral and immoral behaviors may cause asymmetric biases regarding the potential for moral improvement and decline. Specifically, the asymmetric biases in this article are defined as a non-preference for the potential for moral improvement, but an aversion to the potential for moral decline.

The second line of research includes experiments focusing on people’s perception of group morality. For a for-profit company, the improvement of its morality would lead to a decrease in people’s evaluation of its competence [34], which would undoubtedly harm the interests of its employees. If the improvement of corporate morality conflicts with employees’ interests, the employees would reverse their attitude towards the company’s moral improvement [35], resulting in a non-preference for the potential for the company’s moral improvement. As potential employees, applicants may also have a non-preference for the potential for a company’s moral improvement. However, this does not mean that people can accept the decline of corporate morality. A company’s immoral behaviors would engender stakeholders’ anger and contempt, leading to a decline in their evaluation of the company [36–38]. Therefore, this line of research also shows that people may have a non-preference for the potential for a group’s moral improvement, but an aversion to the potential for a group’s moral decline.

### Overview of the present study

To our knowledge, this is the first empirical study on the impact of a group’s potential for moral improvement and decline on talent attraction. Four experiments examined the impact of a group’s potential for moral improvement and decline on individuals’ willingness to join the group.

Experiment 1 examined whether participants preferred the group’s potential for competence or moral improvement; that is, whether they would prefer to join a company with moderate competence/morality but high potential for competence/moral improvement over a company with stably high competence/morality.

Experiment 2 examined whether participants were averse to a group’s potential for competence/moral decline; that is, whether they would be more reluctant to join a company with high competence/morality but high potential for competence/moral decline relative to a company with stably moderate competence/morality.

Experiment 3 directly examined whether participants’ biases regarding the potential for moral improvement and decline were asymmetric. Specifically, we compared participants’ willingness to join companies with high potential for moral improvement, high potential for moral decline, and stable morality.

Experiment 4 set the average moral score of the companies with high potential for moral improvement, high potential for moral decline, and stable morality to be equal, and more strictly evaluated the asymmetric biases regarding the potential for moral improvement and decline.

In our experiments, in order to test the effects of the potential for improvement and decline independently, we adjusted the paradigm of Experiment 3 in Tormala et al.’s study [6] and manipulated the experimental conditions by presenting different historical performance trends. Specifically, we created the potential-for-improvement condition by presenting moderate achievements with an uptrend, the potential-for-decline condition by presenting high achievements with a downtrend, and the control condition by presenting achievements averaging the other two conditions. Thus, if participants preferred the potential for improvement (versus the control condition), this would indicate a preference for the potential for improvement. On the other hand, if they were more averse to the potential for decline (versus the control condition), this would indicate an aversion to the potential for decline.

We hypothesized that individuals would prefer to join a company with moderate competence but high potential for competence improvement relative to a company with stably high competence (Hypothesis 1). However, individuals may have no preference for joining a company with moderate morality but high potential for moral improvement relative to a company with stably high morality (Hypothesis 2). In addition, we proposed that individuals would be more reluctant to join a company with high competence or morality but high potential for competence or moral decline compared with a company with stably moderate competence or morality (Hypothesis 3). More importantly, we expected that individuals’ biases regarding the potential for moral improvement and decline would be asymmetric; that is, individuals would have no preference for joining a company with high potential for moral improvement compared with the control condition, but would be more reluctant to join a company with high potential for moral decline (Hypothesis 4).

## Experiment 1

Experiment 1 examined whether there is a preference for a group’s potential for moral or competence improvement over a group’s high morality or competence.

### Methods

#### Participants

This study was carried out in accordance with the recommendations of the American Psychological Association ethical guidelines. The protocol was approved by the Ethics Committee of the Center for Studies of Social Psychology at Central China Normal University (CSSP-2019012). Before conducting the experimental procedure, all participants were given an informed consent form in accordance with the Declaration of Helsinki. The informed consent form included a brief description about the study, as well as the confidentiality of their data in terms of remaining anonymous in any publication related to this study. It also informed them about their right to withdraw from the study at any time, and included contact information of the researchers so that participants could inquire about any further details of the study.

We used G*Power 3.1 [39] to calculate the desired sample size. We based our power analysis on previous research on the potential preference effects [6], which found an effect size of *f* = 0.24. To observe such an effect with an alpha of .05 and a power of .90, we needed a total sample of 92 participants. Participants were recruited via the Psychology School’s participant pool. Because web surveys are as effective as print surveys [40] and save more resources, we chose to use the online survey platform Sojump (https://www.sojump.com). A total of 92 Chinese adults (19 men and 73 women; *M*_age_ = 20.40 years, *SD* = 2.46) completed the experiment individually online. We prevented the submission of multiple responses from individual participants by restricting access from the same IP address in Experiments 1, 2, 3 and 4. All participants read the informed consent form and agreed to participate in the experiment. They received RMB 3 yuan via WeChat money transfer for their participation.

#### Procedure

**Fig 1:**
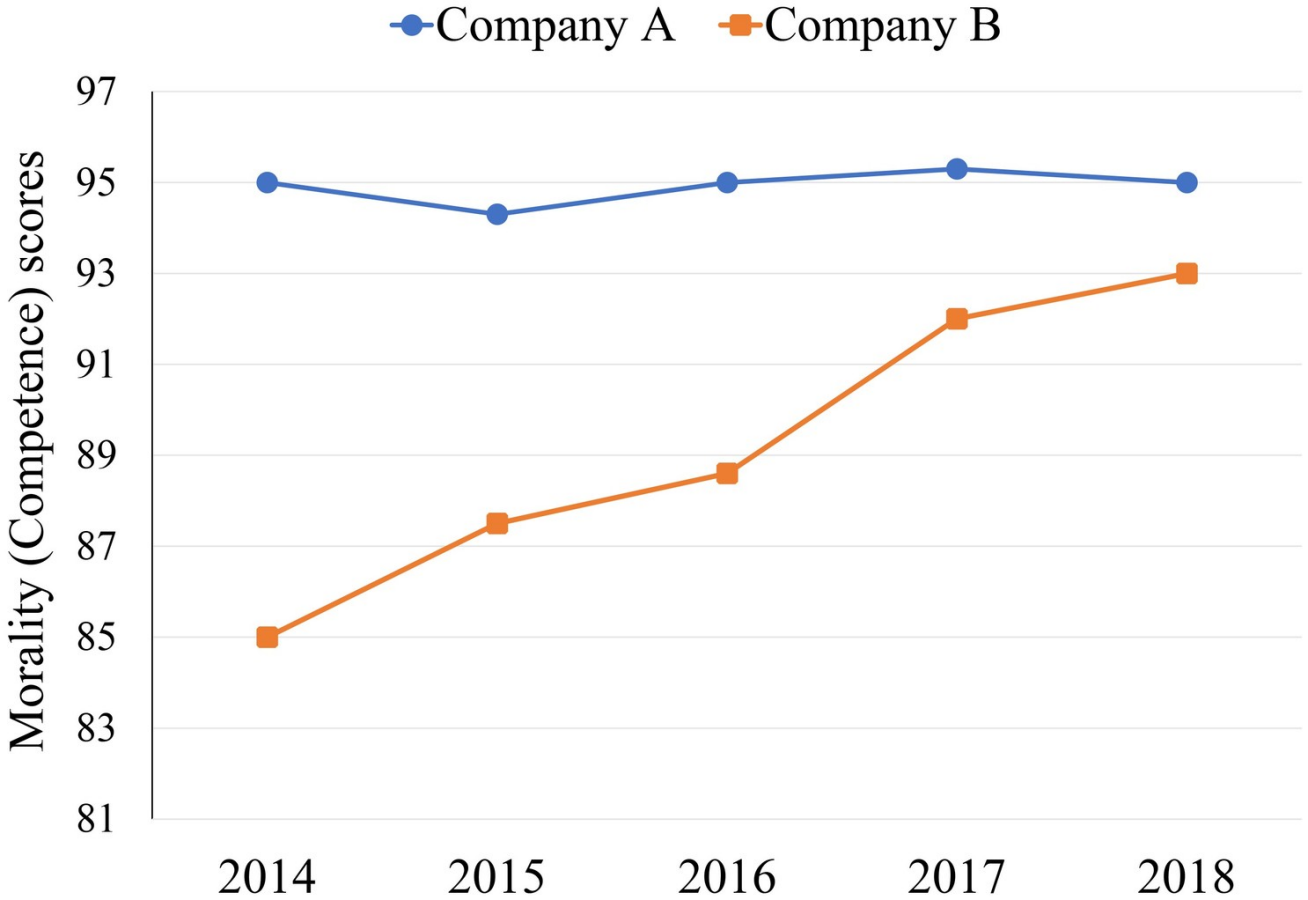
Morality (competence) scores of Company A and Company B from 2014 to 2018 in Experiment 1.

We used a 2 (dimension: morality vs. competence) × 2 (characteristic: stably high performance vs. high potential for improvement) mixed design, with characteristic as a within-participants factor. In the morality (competence) dimension, the opening instructions indicated that participants would receive information on the ranking of two companies’ morality (competence) published by an authoritative and independent research organization. The assessment of company morality (competence) included company ethics and compliance, company citizenship and responsibility, moral culture, company governance, and reputation (business performance, production and procurement ability, global marketing ability, international human resources, innovative research and development ability, and brand building ability). The dimensions of moral evaluation refer to the evaluation criteria of Ethisphere Institute’s annual list of the most ethical enterprises (see https://ethisphere.com/128-worlds-most-ethical-companies-for-2019/). The dimensions of competence evaluation refer to the criteria of Roland Berger and Global Entrepreneur magazine to rank the competitiveness of Chinese enterprises in 2009. Subsequently, participants read that Company A and Company B were two companies with the same level of strength (ethics) in the same industry. Then, they received morality (competence) ranking information from the past 5 years about these two companies on the computer screen. To define which company had stably high performance or high potential for improvement, we structured companies’ morality (competence) scores such that Company A had a stably high performance (95/100 in 2014; 95/100 in 2018), whereas Company B had a moderate performance but a high potential for improvement (85/100 in 2014; 93/100 in 2018). A morality (competence) score of 95 indicated that the company morality (competence) ranked in the top 5% of the industry, and 85 in the top 15%. Participants viewed a graph displaying the morality (competence) scores from 2014 to 2018 (Fig 1).

Below the information about the companies on the same screen, participants were instructed to imagine being applicants and to indicate their willingness to join each company (i.e., If you were an applicant, to what extent would you be willing to join Company A?). Responses to these items were provided on a scale ranging from 1 (*not at all*) to 7 (*very high*). Then, they indicated which company they would like to join (i.e., If you were an applicant, which company would you prefer to join?). Finally, they completed the manipulation check.

### Manipulation check

To measure the effect our manipulation had on companies’ characteristics, participants were asked to select the company with higher performance and that with higher potential for improvement. The manipulation had the expected effect: all participants selected Company B as the company with higher performance, and 91.3% (84 out of 92) of the participants selected Company B as the company with higher potential for improvement.

### Results

**Fig 2:**
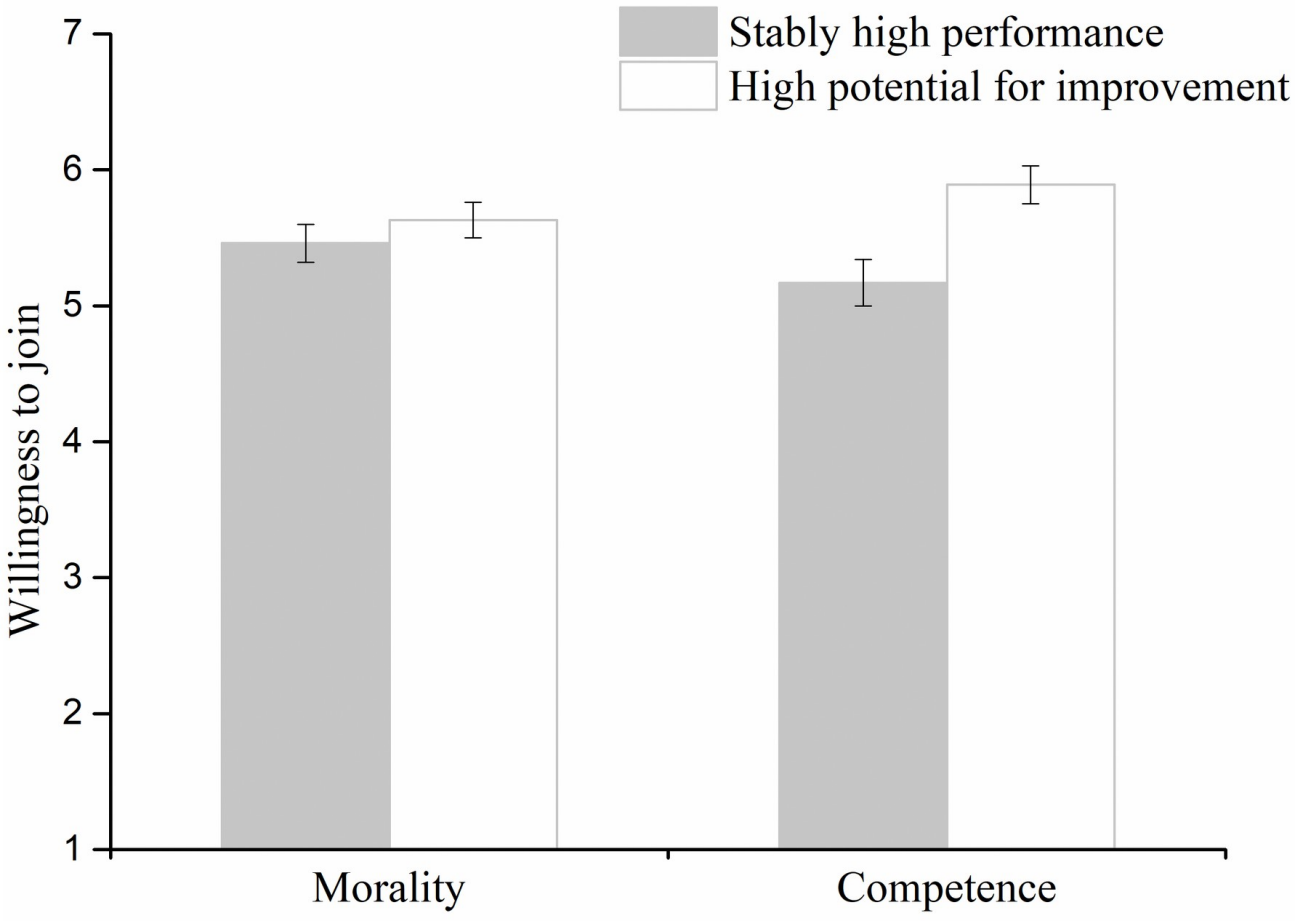
The willingness to join companies with stably high performance and high potential for improvement in morality and competence conditions in Experiment 1. Error bars represent standard errors.

An ANOVA on dimension and characteristic with repeated measures on the latter factor revealed a significant main effect of characteristic, *F*(1, 90) = 9.91, *p* < .01, $\eta_{\text{p}}^{2}=.10$. Participants showed a higher willingness to join the company with high potential for improvement (*M* = 5.76, *SD* = 0.92) than the stably-high-performance company (*M* = 5.32, *SD* = 1.08). Although the effect of the interaction was not significant (*F*(1, 90) = 3.69, *p* = .058, $\eta_{\text{p}}^{2}=.04$), we conducted a planned analysis, which was very important to test our hypothesis. Therefore, we analyzed simple main effects of dimension. In the competence condition, participants showed a higher willingness to join the company with high potential for improvement (*M* = 5.89, *SD* = 0.92; *F*(1, 90) = 12.84, *p* = .001, $\eta_{\text{p}}^{2}=.12$) than the stably-high-performance company (*M* = 5.17, *SD* = 1.18; Fig 2). However, in the morality condition, the willingness to join the company with high potential for improvement (*M* = 5.63, *SD* = 0.90) was not significantly different from the willingness to join the stably-high-performance company (*M* = 5.46, *SD* = 0.96; *F*(1, 90) = 0.75, *p* = .39, $\eta_{\text{p}}^{2}<.01$; Fig 2). The main effect of dimension was not significant (*F*(1, 90) = 0.01, *p* = .94, $\eta_{\text{p}}^{2}<.01$).

In the competence condition, more than half of the participants (36/46) indicated that they preferred to join the company with high potential for improvement (*χ*^2^(1) = 14.70, *p* < .001). However, in the morality condition, only about half of the participants (24/46) reported they preferred to join the company with high potential for improvement (*χ*^2^(1) = 0.09, *p* = .77).

The rating and selection results were consistent. Overall, participants preferred the company with high potential for competence improvement over the company with stably high competence, but they showed no preference for the company with high potential for moral improvement relative to the company with stably high morality.

## Experiment 2

Referring to the manipulation of the potential for improvement in Experiment 1, Experiment 2 examined the aversion to the potential for decline in morality and competence. We presumed that individuals would be more reluctant to join high-performance groups with high potential for moral or competence decline compared with stably-moderate-performance groups.

### Methods

#### Participants

Participants were recruited via the Psychology School’s participant pool. A total of 97 Chinese adults (23 men and 74 women; *M*_age_ = 21.65 years, *SD* = 4.88) completed the experiment individually online through Sojump. All participants read the informed consent form and agreed to participate in the experiment. They received RMB 3 yuan via WeChat money transfer for their participation. We utilized a medium effect size of *f* = 0.24, in line with past work on the potential preference effects [6] and Experiment 1. A sensitivity analysis conducted with G*Power [39] showed that our sample was sufficient to detect medium effects of *f* = 0.26, assuming an alpha of .05 and a power of .90 for an ANOVA with repeated measures (observed correlation among repeated measures, *r* = -0.18).

#### Procedure

**Fig 3:**
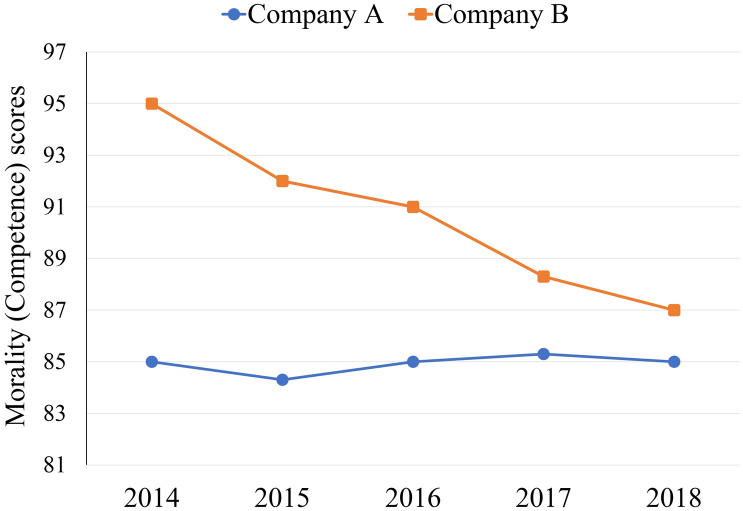
Morality (competence) scores of Company A and Company B from 2014 to 2018 in Experiment 2.

We used a 2 (dimension: morality vs. competence) × 2 (characteristic: stably moderate performance vs. high potential for decline) mixed design, with characteristic as a within-participants factor. The opening instructions were the same as in Experiment 1. Participants received morality (competence) ranking information from the past 5 years about two companies (Company A and Company B) on the computer screen. We structured companies’ morality (competence) scores such that Company A was stably moderate in performance (85/100 in 2014; 85/100 in 2018), whereas Company B was high in performance (95/100 in 2014; 87/100 in 2018) but had a high potential for decline. The morality (competence) scores had the same meaning as in Experiment 1. Participants viewed a graph displaying the morality (competence) scores from 2014 to 2018 (Fig 3).

Below the information about the companies on the same screen, participants rated their willingness to join each company and which company they would like to join (same measures as in Experiment 1). Then, they completed the manipulation check.

### Manipulation check

To measure the effect of our manipulation on characteristic, participants were asked to select the higher performance company and the company with higher potential for decline. The manipulation had the expected effect: all participants selected Company B as the company with higher performance, and 78.4% (76 out of 96) of the participants selected Company B as the company with higher potential for decline.

### Results

**Fig 4:**
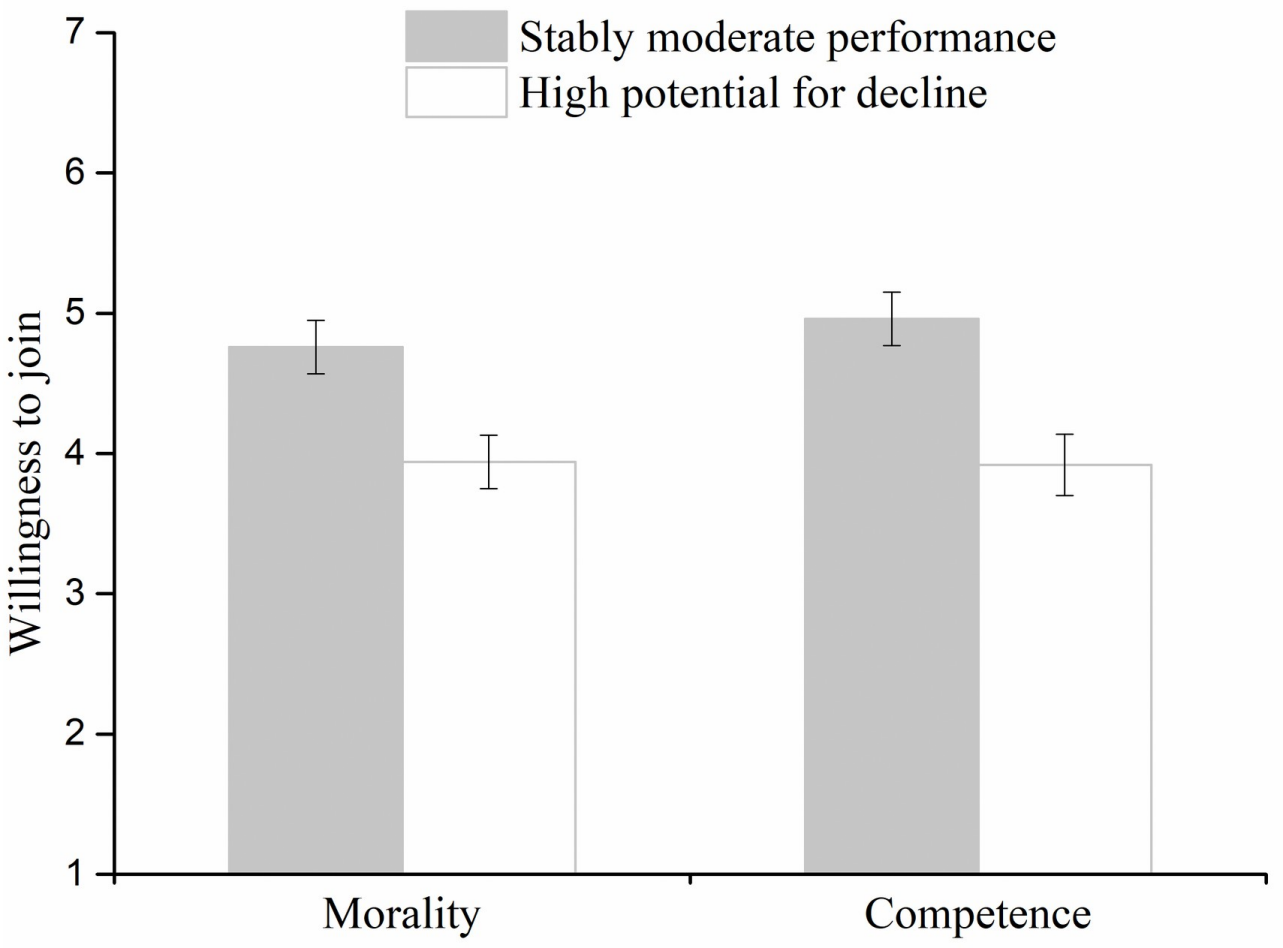
The willingness to join companies with stably moderate performance and high potential for decline in morality and competence dimensions in Experiment 2. Error bars represent standard errors.

An ANOVA on dimension and characteristic with repeated measures on the latter factor revealed a significant main effect of characteristic (*F*(1, 95) = 19.15, *p* < .001, $\eta_{\text{p}}^{2}=.17$). Participants reported a lower willingness to join the company with high potential for decline (*M* = 3.93, *SD* = 1.40) than the company with stable moderate performance *M* = 4.86, *SD* = 1.32). The main effect of dimension (*F*(1, 95) = 0.26, *p* = .61, $\eta_{\text{p}}^{2}<.01$) and the effect of the interaction (*F* (1, 95) = 0.28, *p* = .60, $\eta_{\text{p}}^{2}<.01$), were not significant (Fig 4).

In the competence condition, more than half of the participants (33/48) reported they preferred to join the stably moderate-performance company (*χ*^2^(1) = 6.75, *p* < .01). Similarly, in the morality condition, there was a tendency for more participants to select to join the stably moderate-performance company (30/49) than the high-performance company with high potential for decline (19/49; *χ*^2^(1) = 2.47, *p* = .12).

The rating and selection results were consistent. Overall, both in the morality and competence conditions, individuals were more reluctant to join the high-performance company with high potential for decline than the stably-moderate-performance company.

## Experiment 3

Experiments 1 and 2 implied that the biases regarding the potential for moral improvement and decline may be asymmetric; that is, individuals may not have a preference for the potential for moral improvement, but they may have an aversion to the potential for moral decline. Experiment 3 set three companies (i.e., with high potential for improvement, high potential for decline, and stable performance) to directly investigate individuals’ asymmetric biases regarding a company’s potential for moral improvement and decline.

### Methods

#### Participants

Participants were recruited via the Psychology School’s participant pool. A total of 60 Chinese adults (13 men and 47 women; *M*_age_ = 19.75 years, *SD* = 1.49) completed the experiment individually online through Sojump. All participants read the informed consent form and agreed to participate in the experiment. They received RMB 3 yuan via WeChat money transfer for their participation. Based on Experiments 1 and 2, we utilized a medium effect size of *f* = 0.24. A sensitivity analysis conducted with G*Power [39] showed that our sample was sufficient to detect medium effects of *f* = 0.24, assuming an alpha of .05 and a power of .90 for a within-participants ANOVA (observed correlation among repeated measures, *r* = 0.22).

#### Procedure

**Fig 5:**
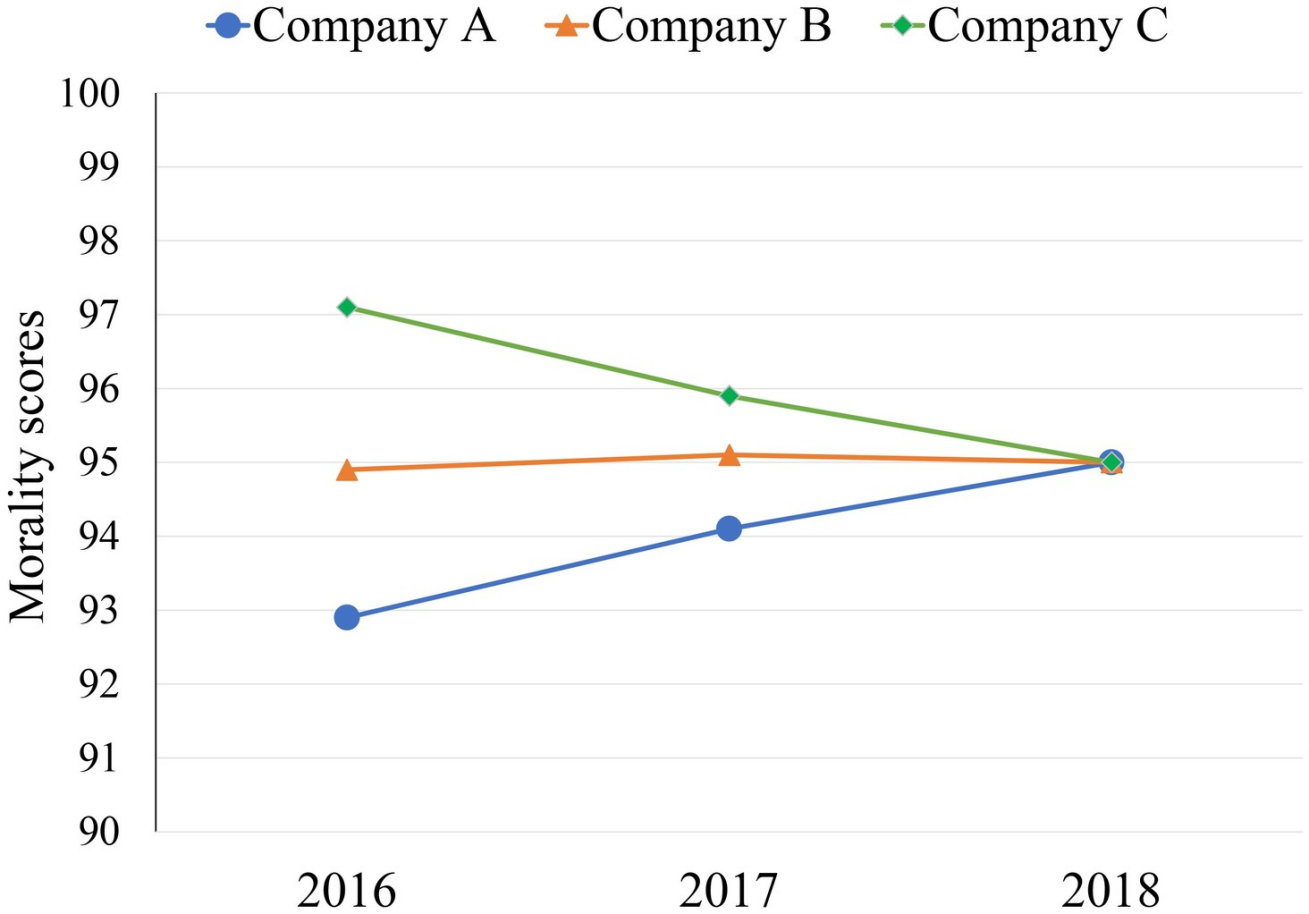
Morality scores of Company A, Company B, and Company C from 2016 to 2018 in Experiment 3.

We used a within-participants design with a 3-level factor (characteristic: high potential for improvement vs. stable performance vs. high potential for decline). The opening instructions were the same as in Experiments 1 and 2. Participants received morality ranking information from the past 3 years about Company A, Company B, and Company C on the computer screen. To define what company had high potential for improvement, stable performance, or high potential for decline, we structured companies’ morality scores such that Company A was moderate in performance (92.9/100 in 2016; 95/100 in 2018) but had high potential for improvement. Company C was high in performance (97.1/100 in 2016; 95/100 in 2018) but had high potential for decline. Company B’s morality scores were in the middle between Company A and Company C and stayed stable (94.9/100 in 2016; 95/100 in 2018). The morality scores had the same meaning as in Experiments 1 and 2. Participants viewed a graph displaying the morality scores from 2016 to 2018 (Fig 5).

Below the information about Company A, Company B, and Company C on the same screen, participants rated their willingness to join each company (same measures as in Experiments 1 and 2) and indicated which company they were willing to join. Then, they completed the manipulation check.

### Manipulation check

To measure the effect of our manipulation on characteristic, participants were asked to rank the potential for improvement (decline) of Company A, Company B, and Company C (i.e., Please rank the companies’ potential for moral improvement (decline) of Company A, Company B, and Company C, and rank the one you think has the highest potential as the first and the one with the lowest potential as the third). The manipulation of the potential for improvement had the expected effect. A Friedman test showed that the ranking of the potential for improvement differed significantly (*χ*^2^(2) = 18.03, *p* < .001). Wilcoxon signed-rank tests showed that Company A (1.58) was ranked higher than Company B (2.07) (*Z* = 3.12, *p* < .01) and Company C (2.35) (*Z* = 3.02, *p* < .01), and Company B was ranked higher than Company C (*Z* = 1.89, *p* = .06), although this result did not reach statistical significance.

The manipulation of the potential for decline also had the expected effect. A Friedman test showed that the rankings of the potential for decline differed significantly (*χ*^2^(2) = 20.13, *p* < .001). Wilcoxon signed-rank tests showed that Company C (1.53) was ranked higher than Company A (2.17) (*Z* = 3.04, *p* < .01) and Company B (2.30) (*Z* = 4.06, *p* < .001), but the rankings of Company A and Company B were not different from each other (*Z* = .94, *p* = .35).

### Results

**Fig 6:**
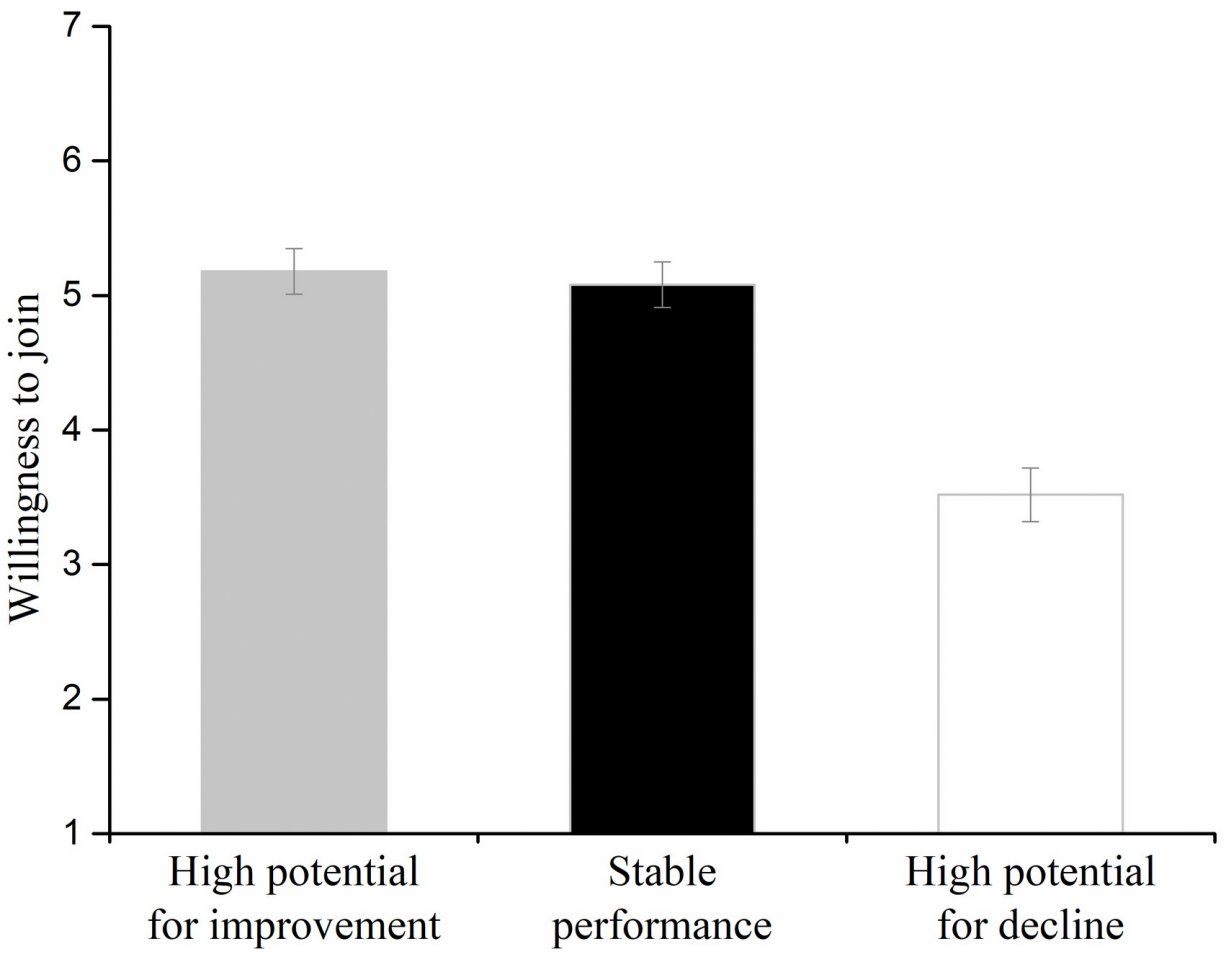
Willingness to join companies with high potential for moral improvement, stably moral performance, and high potential for moral decline in Experiment 3. Error bars represent standard errors.

A within-participants ANOVA revealed a significant effect of characteristic (*F*(2, 118) = 28.66, *p* < .001, $\eta_{\text{p}}^{2}=.33$). Post-hoc comparisons using Fisher’s least significant difference (LSD) test revealed that the willingness to join the company with high potential for improvement (*M* = 5.18, *SD* = 1.35) was not different from the willingness to join the stable-performance company (*M* = 5.08, *SD* = 1.29; Fisher’s LSD: *p* = .63), which were both higher than the willingness to join the high-performance company with high potential for decline (*M* = 3.52, *SD* = 1.51; Fisher’s LSD: *p* < .001; Fig 6).

Participants’ choices between the company with high potential for improvement (30/60) and the stable-performance company (24/60) were not significantly different from each other (*χ*^2^(1) = .67, *p* = .414). However, more participants chose the company with high potential for improvement than the high-performance company with high potential for decline (6/60) (*χ*^2^(1) = 16.00, *p* < .001); moreover, more participants chose the stable-performance company than the high-performance company with high potential for decline (*χ*^2^(1) = 10.08, *p* = .001).

Experiment 3 directly examined the asymmetric biases regarding the potential for moral improvement and decline. The results showed that participants were less willing to join the high-performance company with high potential for decline compared with the stable-performance company. However, participants’ willingness to join the stable-performance company and the company with high potential for improvement did not differ.

## Experiment 4

In order to test the non-preference for the potential for moral improvement more strictly, in Experiment 4, we set the average performance scores of companies with high potential for improvement, high potential for decline, and stable performance to be equal. We examined whether individuals preferred the company with the potential for moral improvement over that with a stable moral performance when their average moral scores were the same.

### Methods

#### Participants

Participants were recruited via the Psychology School’s participant pool. A total of 150 Chinese adults (28 men and 122 women; *M*_age_ = 20.99 years, *SD* = 3.95) completed the experiment individually online through Sojump. All participants read the informed consent form and agreed to participate in the experiment. They received RMB 3 yuan via WeChat money transfer for their participation. Based on Experiments 1, 2 and 3, we utilized a medium effect size of *f* = 0.24. A sensitivity analysis conducted with G*Power [39] showed that our sample was sufficient to detect medium-to-large effects of *f* = 0.29, assuming an alpha of .05 and a power of .90 for a one-way ANOVA.

#### Procedure

**Fig 7:**
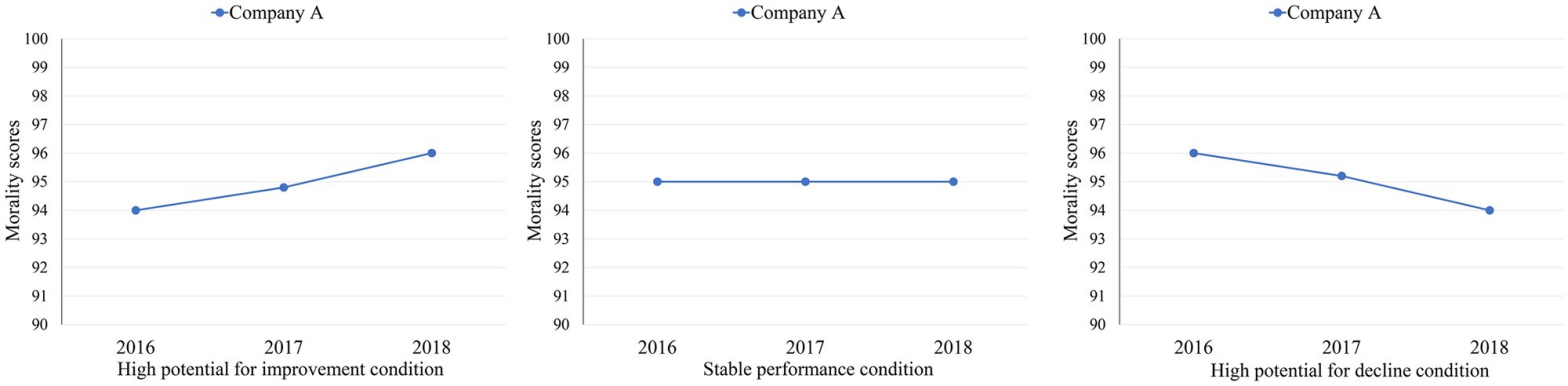
Morality scores of Company A from 2016 to 2018 in high potential for moral improvement, stably moral performance, and high potential for moral decline conditions in Experiment 4.

We used a between-participants design with a 3-level factor (characteristic: high potential for improvement vs. stable performance vs. high potential for decline). The opening instructions were the same as in Experiments 1, 2, and 3. Participants received morality ranking information from the past 3 years about Company A on the computer screen. To define high potential for improvement, stable performance, or high potential for decline, we structured the company’ morality scores. In the first condition, Company A was high in potential for improvement (94/100 in 2016; 96/100 in 2018). In the second condition, the morality scores of Company A were consistent at 95 over the past 3 years. In the third condition, Company A was high in potential for decline (96/100 in 2016; 94/100 in 2018). The morality scores had the same meaning as in Experiments 1, 2, and 3. Participants viewed a graph displaying the morality scores from 2016 to 2018 (Fig 7).

Below the information about Company A on the same screen, participants rated their willingness to join the company (same measures as in Experiments 1, 2, and 3). Then, they completed the manipulation check.

### Manipulation check

Since Experiment 4 mainly focused on the non-preference for the potential for moral improvement, we only conducted the manipulation check on the potential for moral improvement. To measure the effect of our manipulation on characteristic, participants were asked to rate Company A’s potential for improvement (i.e., What do you think is Company A’s potential for moral improvement?) from 1 (very low) to 7 (very high). The manipulation had the expected effect (*F*(2, 147) = 18.44, *p* < .001, $\eta_{\text{p}}^{2}=.20$). Post-hoc comparisons using Fisher’s LSD test revealed that the rating of the company with high potential for improvement (*M* = 5.40, *SD* = 1.28) was higher than that with stable performance (*M* = 4.60, *SD* = 1.18; Fisher’s LSD: *p* = .001), which were both higher than the rating of the company with high potential for decline (*M* = 3.90, *SD* = 1.25; Fisher’s LSD: *p* < .01).

### Results

**Fig 8:**
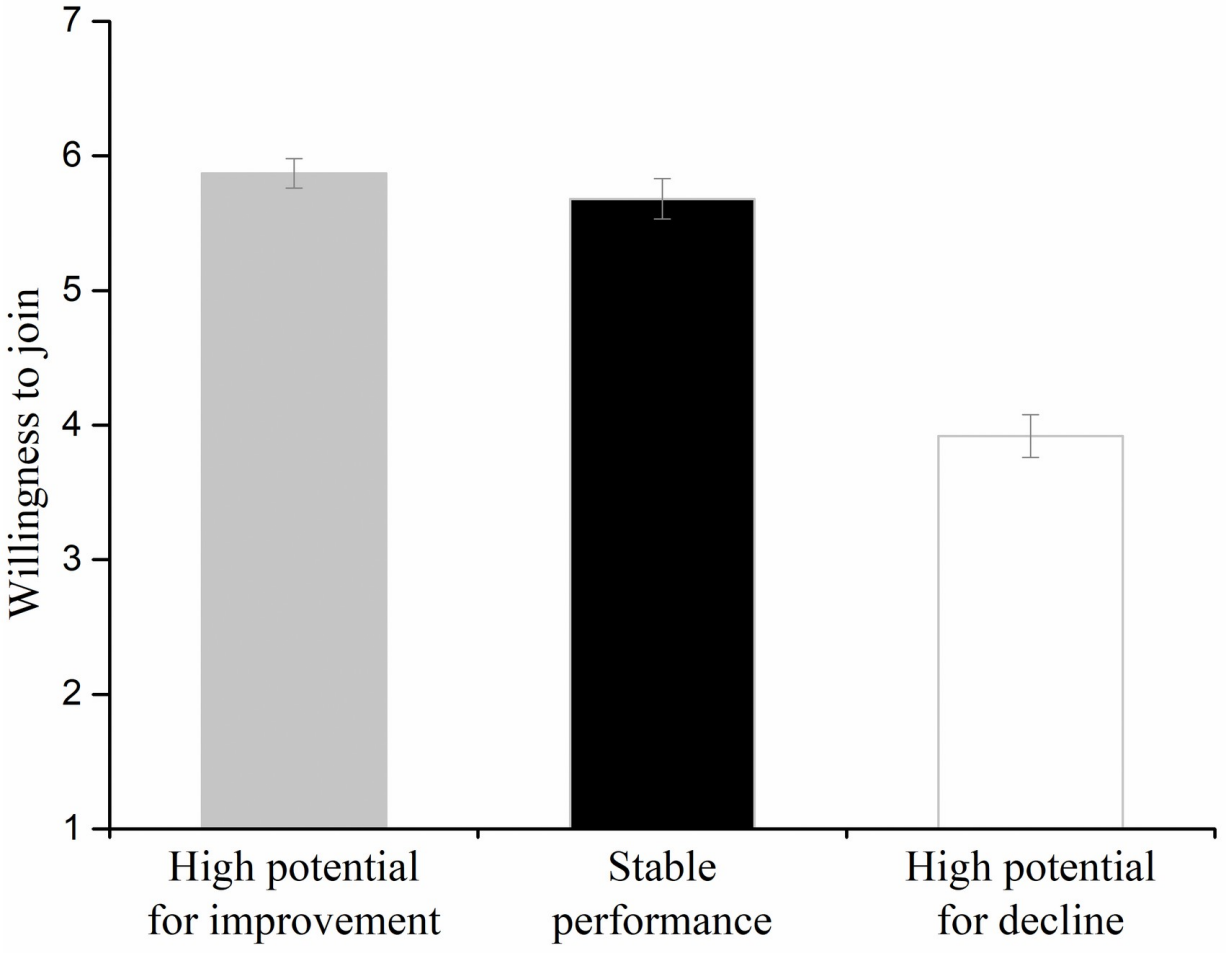
Willingness to join companies with high potential for moral improvement, stably moral performance, and high potential for moral decline in Experiment 4. Error bars represent standard errors.

An ANOVA revealed a significant effect of characteristic (*F*(2, 147) = 53.67, *p* < .001, $\eta_{\text{p}}^{2}=.42$). Post-hoc comparisons using Fisher’s LSD test revealed that the willingness to join the company with high potential for improvement (*M* = 5.96, *SD* = 0.95) was not significantly different from the willingness to join the company with stable performance (*M* = 5.78, *SD* = 1.06; Fisher’s LSD: *p* = .43), which were both higher than the willingness to join the company with high potential for decline (*M* = 3.84, *SD* = 1.36; Fisher’s LSD: *p* < .001; Fig 8).

Experiment 4 set the average morality score of the company with high potential for moral improvement to be consistent with that of the company with stably moral performance. However, participants still showed no preference for the company with high potential for moral improvement. Meanwhile, individuals were more reluctant to join the company with high potential for moral decline than the company with stably moral performance. These results showed that the biases regarding a company’s potential for moral improvement and decline were asymmetric.

## Discussion

Given the importance of corporate morality in attracting talent, it is of great value to examine the impact of a group’s potential for moral improvement and decline on applicants’ willingness to join it. Four experiments showed that individuals had no preference for the potential for moral improvement but were averse to the potential for moral decline. Our findings make several new empirical and theoretical contributions.

Consistent with Hypothesis 1 and previous research on the preference for potential for improvement [6, 7], Experiment 1 found that individuals preferred to join the company with high potential for competence improvement compared with that with stably high competence. Consistent with Hypothesis 2 but inconsistent with previous research [6, 7], the results of Experiments 1, 3, and 4 indicate that individuals had no preference for the company with high potential for moral improvement compared with the company with stably high morality. This inconsistency may have to do with the fact that we assessed the potential for moral improvement, whereas Tormala et al. [6] and Sun et al. [7] focused on the potential for competence improvement. In addition, consistent with Hypothesis 3, Experiment 2 showed that individuals were more reluctant to join a company with high potential for moral or competence decline compared with a company with moderate morality or competence. Importantly, consistent with Hypothesis 4, Experiments 3 and 4 demonstrated that participants’ biases regarding the potential for moral improvement and decline were asymmetric; that is, individuals showed no preference for a company with high potential for moral improvement compared with a company with stably moral performance, but were averse to a company with high potential for moral decline.

### Asymmetric biases regarding moral potential

Morality and competence are two important aspects for people to perceive individuals and groups [10–16]. However, the structure of morality and competence seems to be different. In case of competence, an improvement or decline could be perceived as an upward or downward change in one dimension [41]. Due to the negative bias effect [42], we have reason to infer that if people prefer the potential for competence improvement, then they will also have an aversion to the potential for competence decline. The symmetric biases regarding the potential for competence improvement and decline indirectly indicate that the processing mechanisms of competence improvement and decline are similar.

However, the asymmetric biases regarding the potential for moral improvement and decline indicate that people may process the information of moral improvement and decline differently. Although we manipulated the potential for moral improvement and decline in a similar manner, people may have associated moral improvement and decline with moral and immoral behaviors, respectively. Research shows that people do not force others to become more moral, but they cannot tolerate others becoming more immoral [29]. Consequently, people who perform different degrees of good deeds may be perceived to have the same degree of kindness, but the more bad things they do, the worse they are [31, 32]. In addition, bad actions are often more powerful than good ones [43]. Therefore, although people have no preference for the potential for moral improvement, they are averse to the potential for moral decline.

The situation is a little more subtle for the morality of for-profit companies. Although for-profit companies have made important contributions to social progress, they are stereotypically depicted as immoral and harmful to society [44]. On the contrary, the moral improvement of for-profit companies is considered to hinder their competence [34]. It seems that for-profit companies should pay more attention to the development of their competence. However, no matter how competitive a for-profit company is, any moral decline or scandal may put the company in jeopardy [36–38]. To sum up, the moral improvement of for-profit companies may lead to a decline in people’s evaluation of the companies’ competence, while the companies’ moral decline may lead to people’s worse overall impressions of them. It seems that both the moral improvement and decline of for-profit companies may have negative effects. That situation creates a moral dilemma for for-profit companies. Future research could further test the effect of for-profit corporate moral dilemma.

### Aversion to potential for decline

In Experiment 2, we found that participants were averse to the potential for moral and competence decline. However, the reasons for this aversion may not be the same. Compared with competence, morality is a more essential characteristic of individuals and groups [17, 45]. Compared with negative competence information, negative moral information is viewed as more dominative [11] and diagnostic [46]. The potential for moral decline is likely to have a negative impact on overall impressions about individuals or groups [15], even affecting perceptions of competence [47]. Therefore, the potential for moral decline may result in an overall negative evaluation, leading to an aversion to the potential for decline. However, the potential for competence decline may lead to the anticipation of low future achievements only related to a certain kind of capability, leading to an aversion to the potential for decline.

Moreover, the present research has practical implications for organizations. From the perspective of talent attraction, showing accomplishments and successes is an effective strategy of organizational impression management [48–50]. However, the results of our research suggest that emphasizing the upward potential of the organization may leave a better impression on talent. Meanwhile, the organization should try to avoid creating the impression that it has the potential for moral or competence decline.

### Limitations and future directions

We provided evidence for asymmetric biases regarding the potential for moral improvement and decline. However, before drawing strong conclusions, several limitations should be noted. First, to maintain overall logical consistency and internal validity, the scenarios we set are hypothetical. Although these scenarios ensure the internal validity of the experiment, extra caution is needed when generalizing the conclusions to a broader field. For example, the size and the entitativity of a group can affect people’s moral judgment of the group. Small groups are perceived to be more communal and trustworthy than big ones [51, 52]. Therefore, the moral potential of small companies may have a greater impact than big companies on an applicant’s willingness to join. Perceived entitativity could make people’s moral judgment of the group more extreme. People are more averse to the immoral behaviors of the high entitativity group than the low entitativity group, but they prefer the moral behaviors of the high entitativity group [53, 54]. Therefore, group entitativity may moderate people’s attitude towards the group’s moral improvement and decline. In addition, future research can explore the moderating effect of the characteristics of a company’s morality and the individual motivation on the asymmetric biases regarding a group’s potential for moral improvement and decline. The company’s moral improvement and decline may be caused by the corporate culture or the top-down actions of the company’s leadership. Because the corporate culture is relatively stable, people may deem that a company’s moral improvement and decline driven by leadership is not as lasting as the changes brought about by the corporate culture. Therefore, the corporate culture may have a greater impact on people’s attitude towards a company’s moral improvement and decline than the company’s leadership does. Moreover, applicants who want to change the future of the company may value the company’s moral potential more than those who passively accept the status quo. Overall, testing the boundary conditions of asymmetric biases regarding a group’s moral potential would facilitate more practical application of study findings.

Second, the dependent variables in our study are unified as willingness to join the company, which enables comparison of the results of different experiments. However, it is not clear whether people’s asymmetric biases regarding a group’s moral potential still exist when they need to pay the price for their own decisions. On one hand, research shows the reaction between different dependent variables could be consistent. Specifically, when people express their intention to act towards an individual or a group, hypothetical decisions are similar to their costly behaviors [55, 56]. On the other hand, research shows that real-life decisions are different from hypothetical decisions [57]. In abstract and hedonic decision-making situations, people prefer potential to achievements, while in concrete and utilitarian decision-making situations, people prefer achievements to potential [9, 58]. Future research could use a variety of dependent variables to test the asymmetric biases regarding a group’s moral potential.

Lastly, it is interesting and meaningful to explore how individuals view the process of realizing a group’s potential for improvement and decline. We used linear changes to manipulate the group’s potential in this study. However, group changes may also be non-linear. For example, a change in leadership may lead to huge changes in the overall look of the group. If individuals expect the changes of a group to be non-linear, how will they view the process of realizing the group’s potential? If the process of realizing a group’s potential is unpredictable, will individuals still pay more attention to potentiality than actuality? Answering these questions will promote a deeper understanding of individuals’ attitudes about potentiality and actuality.

## Conclusions and implications

Corporate morality plays an important role in attracting talent, but few experimental studies have focused on the impact of a group’s potential for moral improvement and decline on talent attraction. We investigated this question and found that individuals have asymmetric biases regarding a group’s potential for moral improvement and decline; that is, they are averse to the potential for moral decline but have a non-preference for the potential for moral improvement. On the other hand, individuals have symmetric biases regarding a group’s potential for competence improvement and decline; thus, they are averse to the potential for competence decline and have a preference for the potential for competence improvement. Our study suggests that besides their moral and competence achievements, groups could also emphasize their potential for moral and competence improvement to attract talent. The exploration of the boundary conditions of the asymmetric biases regarding a group’s moral potential and individuals’ views about the process of realizing a group’s potential may be fruitful research directions in the future.
