# Supplementary material for: The preference for potential in competence, not in morality: Asymmetric biases regarding a group's potential for moral improvement and decline
Source: PLoS One. 2020 Aug 4;15(8):e0236748. doi: 10.1371/journal.pone.0236748 (PMC7402473; doi:10.1371/journal.pone.0236748)
Supplement: Attachment — Submitted filename: Response_to_Reviewers.docx [file pone.0236748.s001.docx]

*Dear Editor and Reviewers,*

*Thank you for your letter and the reviewers’ comments concerning our manuscript entitled “The preference for potential in competence, not in morality: Asymmetric biases regarding a group’s potential for moral improvement and decline” (ID: PONE-D-20-05265). Those comments are all very valuable and helpful for us to revise and improve the manuscript, and also have important guiding significance for our research. We studied the comments carefully and have made corrections, hoping to meet your approval. The main corrections in the manuscript and the responds to the reviewers’ comments are as following:*

**Responds to the reviewers’ comments**

**Reviewer #1: Review PONE-D-20-05265**

**The authors test whether people preferred groups depending on whether their morality versus competence was expected to improve versus decline. The question is interesting per se, and the authors offers 4 experiments that test this question.**

*Response: Thank you for your approval of the research issue.*

**I see several avenues for improvement of the manuscript:**

**In the opening example, it would be good to be a bit more concrete. How would someone know that a company is expected to decline in morality in the real world, what would indicate that?**

*Response: Thank you for your comments. In the real world, people can learn about a company’s morality situation by checking the assessment released by rating agencies. For example, Ethisphere Institute rates the most ethical companies in the world every recent year. If a company’s moral ranking has declined linearly in recent years, people may predict that the company’s morality would continue to decline in the future. The corresponding changes in our manuscript are as follows.*

Corporate morality plays an important role in attracting talent [1-5]. Imagine that an authoritative rating agency measured the corporate morality of almost all enterprises in an industry and published the results. You are an applicant who checked the morality rankings of three companies you have been interested in over the past five years, and found that one ranks moderately but has high potential for improvement, one ranks high but has high potential for decline, and one ranks between the other two companies and remains stable. Which company would you like the most? Which company would you like the least?

*(please see changes on page 2)*

**The authors should make clearer whether they expect the same patterns found for the morality and competence of individuals to also hold for the qualities of organizations. This assumption is now somewhat implicit in the theory part, but it should be made clearer by flagging which cited literature pertains to individuals versus organizations in the theoretical introduction. See Critcher & Dunning 2014 or Skowronski 2002 for differences and similarities in the perception of groups versus individuals.**

*Response: Thank you for your comments. In the part of the theoretical review of the symmetric biases regarding competence potential, we explained the similarities and differences in people’s processing of individual and group information. In some ways, people’s perception of the individual and the group is different. For example, compared with the group, people judge the individual more positively. However, in the face of linear development trend, people’s expectations for the future of individuals and groups are similar. In addition, people give more value to future achievements than current achievements. Therefore, people may prefer the individual’s and group’s potential for improvement in terms of competence, and have an aversion to their corresponding potential for decline. We clearly pointed out that the symmetric biases regarding the potential of individual’s and group’s competence both exist. The corresponding changes in our manuscript are as follows.*

Although a preference for the potential for individual competence improvement and an aversion to the potential for individual competence decline were mixed in previous studies, both these biases may exist when people process individual and group information. In some cases, a person’s processing of similar individual and group information will produce different results. For example, compared with groups, people judge the individuals more positively [22]. The negativity bias in honesty judgments and the positivity bias in intelligence judgments are more diminutive in trait judgments of groups than in trait judgments of individuals [23]. However, when facing a linear development trend of individuals, groups, and things, people tend to predict that the linear trend will continue into the future [24-26]. Therefore, when compared with high achievements with a horizontal trend, moderate achievements with the potential for improvement may lead to higher expected future achievements. In addition, individuals generally assign higher values to future achievements than to past achievements [27, 28]. Hence, there may be a preference for individuals and groups with a potential for improvement over those with stable high achievements. Similarly, compared with moderate achievements with a horizontal trend, high achievements with a potential for decline may lead to lower expected future achievements, resulting in an aversion to the potential for decline.

*(please see changes on page 4-5)*

*In the part of the theoretical review of the asymmetric biases regarding moral potential, following he the suggestions of Reviewer #2, we deleted the literature review on identity judgment and replaced it with literature review on how people perceive group morality. The corresponding changes are as follows.*

The second line of research includes experiments focusing on people’s perception of group morality. For a for-profit company, the improvement of its morality would lead to a decrease in people’s evaluation of its competence [34], which would undoubtedly harm the interests of its employees. If the improvement of corporate morality conflicts with employees’ interests, the employees would reverse their attitude towards the company’s moral improvement [35], resulting in a non-preference for the potential for the company’s moral improvement. As potential employees, applicants may also have a non-preference for the potential for a company’s moral improvement. However, this does not mean that people can accept the decline of corporate morality. A company’s immoral behaviors would engender stakeholders’ anger and contempt, leading to a decline in their evaluation of the company [36-38]. Therefore, this line of research also shows that people may have a non-preference for the potential for a group’s moral improvement, but an aversion to the potential for a group’s moral decline.

*(please see changes on page 6).*

**The authors should mention in the abstract that they conducted 4 studies.**

*Response: Thank you for your suggestion. In the abstract, we make it clear that 4 experiments have been done in the research. The corresponding changes are as follows.*

We conducted four experiments with 399 Chinese participants who rated their willingness to join groups with potential for improvement, potential for decline, or stability in terms of morality or competence.

*(please see changes on page 2).*

**In the concrete experiments, the scenarios are very hypothetical. It’s not clear what size the companies would have, in which role the participants imagined to join, and whether they’d imagine any potential to change the company’s morality versus competence. This assessment would probably be different whether someone expects to join a 5-people start-up versus a large company in a minor role. See for example La Macchia, Louis, Hornsey & Leonardelli 2016 on relevant differences between the morality of small and large groups. The authors should discuss or test whether small versus large groups would be some boundary condition.**

*Response: Thank you for your comments. In the discussion section, we discussed how the hypothetical scenarios affect the internal and external validity of the research. Although the hypothetical scenarios are conducive to the internal consistency of the whole research logic, it is not conducive to the generalization of conclusions and the exploration of boundary conditions. We discussed the possible moderating effects of company size and applicants’ motivation on the asymmetric biases regarding groups’ moral potential. The corresponding changes are as follows.*

First, to maintain overall logical consistency and internal validity, the scenarios we set are hypothetical. Although these scenarios ensure the internal validity of the experiment, extra caution is needed when generalizing the conclusions to a broader field. For example, the size of a group can affect people’s moral judgment of the group. Small groups are perceived to be more communal and trustworthy than big ones [50, 51]. Therefore, the moral potential of small companies may have a greater impact on an applicant’s willingness to join than for big companies. In addition, future research can explore the moderate effect of individual motivation on group potential preference. Applicants who want to change the future of the company may value the company’s moral and competence potential more than those who passively accept the status quo. Overall, testing the boundary conditions of asymmetric biases regarding a group’s moral potential and symmetrical biases with regards to its competence potential would facilitate more practical application of study findings.

*(please see changes on page 25).*

**The terminology is inconsistent at times. In the Pilot study, the authors talk about warmth instead of morality, but these are not the same thing. This needs to be clarified or the study needs to be replaced.**

*Response: Thank you for your comments. Warmth assessed in the pilot experiment is indeed inconsistent with the terminology of the whole study. In view of the fact that the deletion of the pilot experiment has no effect on the logic and the integrity of the results of the whole study, we decided to delete the pilot experiment to maintain the consistency of the research terminology and logic.*

**The mechanism underlying the asymmetry between morality and competence should be explained better. Is it that for morality, there is some sort of threshold, where once this is reached, more is not better? In contrast, for competence, more is always better? This should at the very least be elaborated on in the Discussion. The section on Asymmetry is quire repetitive, it would be good to raise some more substantial points here.**

*Response: Thank you for your comments. The section on asymmetry really does not give a more in-depth explanation of the mechanism behind asymmetry, so we rewrote all the contents of this section. As you said, the reason why moral improvement does not lead to higher evaluation may be that there is a sort of threshold in the evaluation of morality. Specifically, the threshold may divide morality into two dimensions: prescriptive morality and proscriptive morality. Prescriptive morality is sensitive to positive outcomes, based on activation, and focuses on what we should do. Whereas proscriptive morality is sensitive to negative outcomes, based on inhibition, and focuses on what we should not do. The improvement of prescriptive morality may not lead to higher evaluation, while the decline of proscriptive morality may lead to lower evaluation. Therefore, people do not prefer the potential for moral improvement, but are averse to the potential for moral decline.*

*However, there may be no such threshold in the evaluation of competence. The change of competence could be perceived as change in one dimension. The improvement of competence leads to higher evaluation, while the decline of competence leads to lower evaluation. The corresponding changes are as follows.*

Morality and competence are two important aspects for people to perceive individuals and groups [10-16]. However, the structure of morality and competence seems to be different. In case of competence, an improvement or decline could be perceived as an upward or downward change in one dimension [41]. Due to the negative bias effect [42], we have reason to infer that if people prefer the potential for competence improvement, then they will also have an aversion to the potential for competence decline. The symmetric biases regarding the potential for competence improvement and decline indirectly indicate that the processing mechanisms of competence improvement and decline are similar.

However, the improvement and decline of morality may correspond to different dimensions, such as prescriptive and proscriptive morality, respectively [29]. Prescriptive morality is sensitive to positive outcomes, based on activation, and focuses on what we should do; whereas, proscriptive morality is sensitive to negative outcomes, based on inhibition, and focuses on what we should not do. An interesting result of this distinction is that people who perform different degrees of good deeds may be perceived to have the same degree of kindness, but the more bad things they do, the worse they are [31, 32]. Therefore, although people are averse to the potential for moral decline, they have no preference for the potential for moral improvement.

*(please see changes on page 23)*

*We also discussed the possible mechanism behind people’s asymmetric biases regarding the moral potential of for-profit companies. The corresponding changes are as follows.*

The situation is a little more subtle for the morality of for-profit companies. Although for-profit companies have made important contributions to social progress, they are stereotypically depicted as immoral and harmful to society [43]. On the contrary, the moral improvement of for-profit companies is considered to hinder their competence [34]. It seems that for-profit companies should pay more attention to the development of their competence. However, no matter how competitive a for-profit company is, any moral decline or scandal may put the company in jeopardy [36-38]. It seems that the morality of for-profit companies should neither improve nor decline. That situation creates a moral dilemma for for-profit companies. Future research could further test the effect of for-profit corporate moral dilemma.

*(please see changes on page 23-24)*

**Experiment 3 seems somewhat redundant. It would be nice to show a behavioral consequence of the effect, eg willingness to join groups when the outcome is less hypothetical and more personally relevant. Alternatively, participants could be asked to bet some small amount on the group. At the very least, it should be discussed in the limitations in light of existing work that shows similar effects for hypothetical versus costly behavior in performance vs. morality perception (Cone & Ferguson, 2015; Steinmetz, Toure-Tillery, & Fishbach, 2020).**

*Response: Thank you for your comments. Based on our current resources and limited time, we are sorry that we may not be able to complete the new experiment. But your suggestion is very instructive to the future research. In the section of limitations and future directions, we discussed the influence of the diversity of dependent variables on the internal and external validity of the study.*

*On the one hand, according to the literature you provided (Cone & Ferguson, 2015; Steinmetz, Toure-Tillery, & Fishbach, 2020), the hypothetical decision is consistent with the decision that needs to pay the price. People’s hypothetical decisions in situations where there is no need to pay a price could be extended to costly behaviors. On the other hand, research shows that hypothetical decisions are different from real-life decisions. Moreover, the decision-making situation could moderate the potential preference effect. Specifically, in abstract and hedonic decision-making situations, people prefer potential to achievements, while in concrete and utilitarian decision-making situations, people prefer achievements to potential. It is not clear whether the decision-making situation could moderate the asymmetric biases regarding group’s moral potential. This issue is very interesting and could be explored more deeply in future research. The corresponding contents in the manuscript are as follows.*

Second, the dependent variables in our study are unified as willingness to join the company, which enables comparison of the results of different experiments. However, it is not clear whether people’s asymmetric biases regarding a group’s moral potential still exist when they need to pay the price for their own decisions. On one hand, research shows the reaction between different dependent variables could be consistent. Specifically, when people express their intention to act towards an individual or a group, hypothetical decisions are similar to their costly behaviors [52, 53]. On the other hand, research shows that real-life decisions are different from hypothetical decisions [54]. In abstract and hedonic decision-making situations, people prefer potential to achievements, while in concrete and utilitarian decision-making situations, people prefer achievements to potential [9, 55]. Future research could use a variety of dependent variables to test the asymmetric biases regarding a group’s moral potential.

*(please see changes on page 25-26).*

**Reviewer #2: The manuscript was generally well-written. The authors presented four rigorous experiments, illustrating people’s (as)symmetric preference for moral-/competence-characterized groups. The line of reasoning was comprehensible, and the experiments were clearly communicated.**

*Response: Thank you for your approval of the research problem and the whole research logic.*

**Despite so, a few remaining questions can be better addressed, specifically as below:**

**1. In the authors’ reasoning about why people demonstrate asymmetric biases in moral improvement versus decline, a core concept “insensitivity” emerged. What does “insensitivity” mean? People are less capable to distinguish? OR less motivated to react to the stimuli? The exact implication of this phrase is somehow vague, and is not fully convincing when referring to the data. For example:**

**1a. In reasoning of “insensitivity”, on page 5, the first paragraph presented empirical evidence on how people are less likely to distinguish morally good deeds than morally bad deeds, while the second paragraph demonstrated studies showing that people are less likely to rely on morally positive than negative information to determine their overall impression. In at least these two examples, “insensitivity” implies different things.**

**1b. When looking at the actual data, people can perfectly distinguish which companies are improving, stable, and declining (according to the manipulation check). So, it seems to me that people just care less about morality in its upward than downward trend.**

**Concerning the critical role of “insensitivity” in the line of reasoning, this concept deserves better clarification, or replacement with more appropriate expressions (valuation, weight, etc.).**

*Response: Thank you for your comments. As you said, the concept of “insensitivity” doesn’t explain the asymmetric biases very well. In the experimental manipulation, people can distinguish between the improvement and decline of group morality in different degrees. They just don’t seem to care too much about the improvement of group morality. In view of the references you have provided (Janoff-Bulman, Sheikh & Hepp, 2009), the improvement and decline of group morality are likely to correspond to prescriptive and proscriptive morality, respectively. People do not have mandatory requirements for prescriptive morality, so different degrees of moral improvement are acceptable. However, people can’t accept the violation of proscriptive morality. Therefore, following your suggestion, we changed the term “insensitivity” to “evaluation”. That is, the evaluations of the improvement and decline of morality are asymmetric. The corresponding changes are as follows.*

Individuals may prefer the potential for a group’s competence improvement and experience aversion to the potential for a group’s competence decline. However, at least two lines of research suggest that individuals may have a non-preference for the potential for a group’s moral improvement, but an aversion to the potential for a group’s moral decline. The first line includes experiments evaluating beliefs about others’ moral improvement and decline, which showed that people’s evaluations of moral and immoral behaviors are asymmetric [29-33]. For example, Klein and Epley [33] described a situation in which an accomplished professor who received an $80,000-grant for his research decided to donate $10,000 to a nonprofit institution dedicated to research on poverty; this action was rated as “nice” as donating the whole sum of $80,000. However, if this accomplished professor found a bag containing $80,000 and took all $80,000 for himself, this action was rated as less “nice” than taking $10,000 for himself and returning the rest to the police. Because moral behaviors are not mandatory, people’s evaluations of the growing good deeds will not increase in proportion. However, owing to low tolerance of immoral behaviors, sanctions against increasing immoral behaviors will become more and more prominent [29]. Therefore, these asymmetric evaluations of moral and immoral behaviors may cause asymmetric biases regarding the potential for moral improvement and decline; that is, individuals may have a non-preference for the potential for moral improvement, but an aversion to the potential for moral decline.

*(please see changes on page 5-6)*

*We also discussed this issue in the discussion section. The symmetric biases regarding competence potential may be due to that the improvement and decline of competence are in one dimension, whereas the asymmetric biases regarding moral potential may be due to that the improvement and decline of morality correspond to two dimensions of morality respectively. It may be that the two dimensions of morality have different attributes that lead to people’s asymmetric biases regarding the potential of moral improvement and decline. The corresponding contents are as follows.*

Morality and competence are two important aspects for people to perceive individuals and groups [10-16]. However, the structure of morality and competence seems to be different. In case of competence, an improvement or decline could be perceived as an upward or downward change in one dimension [41]. Due to the negative bias effect [42], we have reason to infer that if people prefer the potential for competence improvement, then they will also have an aversion to the potential for competence decline. The symmetric biases regarding the potential for competence improvement and decline indirectly indicate that the processing mechanisms of competence improvement and decline are similar.

However, the improvement and decline of morality may correspond to different dimensions, such as prescriptive and proscriptive morality, respectively [29]. Prescriptive morality is sensitive to positive outcomes, based on activation, and focuses on what we should do; whereas, proscriptive morality is sensitive to negative outcomes, based on inhibition, and focuses on what we should not do. An interesting result of this distinction is that people who perform different degrees of good deeds may be perceived to have the same degree of kindness, but the more bad things they do, the worse they are [31, 32]. Therefore, although people are averse to the potential for moral decline, they have no preference for the potential for moral improvement.

*(please see changes on page 23)*

**2. The focal research question (as presented in the DV, “If you were an applicant, to what extent would you be willing to join Company …?”) focuses on people’s intended interaction with group (especially business-framed group emphasizing competence) rather than individual. However, most of the literature overemphasized stereotype content influences on individual perception and interaction. It may be plausible to posit that, for example, people are less willing to join morally improving companies since moral improvement of companies can compromise its competence (Aaker, Vohs, & Mogilner, 2010) and efficiencies in promoting applicants’ self-benefits. And morally positive targets can be perceived as less moral when they threaten self-interest (Melnikoff & Bailey, 2018). OR, negative moral changes usually stimulate more sanctions than positive ones (Janoff-Bulman, Sheikh, & Hepp, 2009), which also pose higher influence on potential applicants’ self-interest.**

**2a. Thus, I expect that the authors can provide more reasoning and literature regarding why people interact with companies and organizations (especially in business-oriented mindset or situations; rather than individuals) depending on their moral-related stereotype contents.**

**2b. Relatedly, page 5 line 112, it seems irrelevant to discuss morality and identity judgment. Self-concept and other-perception are different domains and can have different cognitive mechanisms.**

*Response: Thank you for your insightful ideas on this study. We reviewed the literature on the evaluations of individuals to companies, and proposed that there may be a dilemma in people’s attitudes towards for-profit companies’ morality. The purpose of a for-profit company is to make bigger profits, and people may join the job to get a higher salary. To this end, for-profit companies need to constantly improve their competence. However, the improvement of corporate morality is likely to reduce people’s evaluation of the company’s competence, thus affecting the interests of its employees. As potential employees, applicants are likely to lower their evaluation of the company’s moral improvement. Meanwhile, the company’s moral decline would undoubtedly lower people’s evaluation of the company. Therefore, there may be a moral dilemma for for-profit companies. The corresponding changes in the section of introduction are as follow.*

The second line of research includes experiments focusing on people’s perception of group morality. For a for-profit company, the improvement of its morality would lead to a decrease in people’s evaluation of its competence [34], which would undoubtedly harm the interests of its employees. If the improvement of corporate morality conflicts with employees’ interests, the employees would reverse their attitude towards the company’s moral improvement [35], resulting in a non-preference for the potential for the company’s moral improvement. As potential employees, applicants may also have a non-preference for the potential for a company’s moral improvement. However, this does not mean that people can accept the decline of corporate morality. A company’s immoral behaviors would engender stakeholders’ anger and contempt, leading to a decline in their evaluation of the company [36-38]. Therefore, this line of research also shows that people may have a non-preference for the potential for a group’s moral improvement, but an aversion to the potential for a group’s moral decline.

*(please see changes on page 6)*

*The corresponding changes in the section of discussion are as follows.*

The situation is a little more subtle for the morality of for-profit companies. Although for-profit companies have made important contributions to social progress, they are stereotypically depicted as immoral and harmful to society [43]. On the contrary, the moral improvement of for-profit companies is considered to hinder their competence [34]. It seems that for-profit companies should pay more attention to the development of their competence. However, no matter how competitive a for-profit company is, any moral decline or scandal may put the company in jeopardy [36-38]. It seems that the morality of for-profit companies should neither improve nor decline. That situation creates a moral dilemma for for-profit companies. Future research could further test the effect of for-profit corporate moral dilemma.

*(please see changes on page 23-24)*

*We agree with you that the relationship between identity judgment and the purpose of this study is weak. Although this line of research on identity judgment supports people’s emphasis on the moral decline in judging other’s identity, it focuses on the cognition of identity judgment and has a weak relationship with the perception of group value. Therefore, we deleted the literature review and the related discussions about identity judgment in the manuscript.*

**3. Two minor issues:**

**3a. Sample size across the studies should be sufficient; however, design in Study 4 (between-subjects) was quite different from previous ones (within-subjects). At least, a sensitivity power analysis should be conducted to justify the sample of Study 4.**

*Response: Thank you for your approval of the sample size of the experiments. We have modified the calculation parameters and reference of effect size of a priori analysis in experiment 1. The corresponding contents are as follows.*

Experiment 1

We used G*Power 3.1 [39] to calculate the desired sample size. We based our power analysis on previous research on the potential preference effects [6], which found an effect size of *f*= 0.24. To observe such an effect with an alpha of .05 and a power of .90, we needed a total sample of 92 participants.

*(please see changes on page 9)*

*In experiment 2, 3 and 4, we conducted a sensitivity power analysis, which shows that the sample sizes are sufficient to detect medium effect sizes. The corresponding contents are as follows.*

Experiment 2

We utilized a medium effect size of *f*=0.24, in line with past work on the potential preference effects [6] and Experiment 1. A sensitivity analysis conducted with G*Power [39] showed that our sample was sufficient to detect medium effects of *f*=0.26, assuming an alpha of .05 and a power of .90 for an ANOVA with repeated measures (observed correlation among repeated measures, *r*= -0.18).

*(please see changes on page 13)*

Experiment 3

Based on Experiments 1 and 2, we utilized a medium effect size of *f*=0.24. A sensitivity analysis conducted with G*Power [39] showed that our sample was sufficient to detect medium effects of *f*=0.24, assuming an alpha of .05 and a power of .90 for a within-participants ANOVA (observed correlation among repeated measures, *r*=0.22).

*(please see changes on page 16)*

Experiment 4

Based on Experiments 1, 2 and 3, we utilized a medium effect size of *f*=0.24. A sensitivity analysis conducted with G*Power [39] showed that our sample was sufficient to detect medium-to-large effects of *f*=0.29, assuming an alpha of .05 and a power of .90 for a one-way ANOVA.

*(please see changes on page 19).*

**3b. On page 22, why did the authors only examine potential for moral improvement in the manipulation check? The missing check of moral decline is not equivalent to that in previous studies.**

*Response: Thank you for your comments. In experiment 4, we only set up the manipulation check on the potential for moral improvement, but did not test the manipulation on the potential for moral decline, which is really different from experiment 3. We did this because in experiment 4, we mainly wanted to test whether people still have no preference for the potential for moral improvement in more extreme situations.*

*In this situation, the average moral levels of the three companies are the same, and in the last year, the moral level of the company with potential for improvement has even exceeded that of the company with stable morality. However, the dual advantages of the company with potential for moral improvement (high morality in the recent year and high potential for moral improvement) have not made people prefer it.*

*In this situation, however, the company with potential for moral decline have double disadvantages (low morality in the recent year and high potential for moral decline). In this case, people would undoubtedly be more averse to the company with potential for moral decline. Therefore, we only set up manipulation check on the potential for moral improvement.*

Since Experiment 4 mainly focused on the non-preference for the potential for moral improvement, we only conducted the manipulation check on the potential for moral improvement. To measure the effect of our manipulation on characteristic, participants were asked to rate Company A’s potential for improvement (i.e., What do you think is Company A’s potential for moral improvement?) from 1 (very low) to 7 (very high).

*(please see changes on page 20-21).*

**References**

**Aaker, J., Vohs, K. D., & Mogilner, C. (2010). Nonprofits are seen as warm and for-profits as competent: Firm stereotypes matter. Journal of Consumer Research, 37(2), 224-237.**

**Janoff-Bulman, R., Sheikh, S., & Hepp, S. (2009). Proscriptive versus prescriptive morality: Two faces of moral regulation. Journal of personality and social psychology, 96(3), 521.**

**Melnikoff, D. E., & Bailey, A. H. (2018). Preferences for moral vs. immoral traits in others are conditional. Proceedings of the National Academy of Sciences, 115(4), E592-E600.**

**Reviewer #3: There are many issues with this paper.**

**First and foremost, equating 'warmth' with 'morality' is problematic, and the paper jumps from intent in the introduction to 'warmth' to 'strength (ethics)' to 'morality (competence)'. This is very confusing as it seems that the authors keep switching what they are writing about and are mixing very different things.**

*Response: Thank you for your comments. We admit that it is not appropriate to measure “warmth” rather than “morality” in pilot experiment. In view of the weak logical relationship between the pilot experiment and the whole study, deleting the pilot experiment has no effect on the logic of the whole study and the integrity of the conclusions, we deleted the pilot experiment.*

**Second, the paper does not provide crucial information regarding the recruitment methods:**

**were there incentives given to participants?**

**did they complete informed consent?**

**what efforts were taken to ensure participant privacy and data protection?**

**what is the literature on the characteristics and reliability of Sojump respondents (e.g., compared to convenience samples of students or to representative samples)?**

*Response: Thank you for your comments. We added information about experimental remuneration and informed consent in the manuscript. The participants were recruited via the participant pool of the School of Psychology. The participants in the participant pool includes college students, graduate students and some social personnel. Participants who completed the experiment will receive 3 yuan for their participation. Before starting the experiment, the participants would read the informed consent form, and only with the consent will we collect the data of the participants. Since we did not record the specific information of the subjects, such as name and address, the participants were anonymous to us. Before the start of the experiment, we will also declare that we will not disclose the participants’ personal information, and all the data will only be used for scientific research and analysis. The corresponding changes are as follows.*

Experiment 1

This study was carried out in accordance with the recommendations of the American Psychological Association ethical guidelines. The protocol was approved by the Ethics Committee of the Center for Studies of Social Psychology at Central China Normal University (CSSP-2019012). Before conducting the experimental procedure, all participants were given an informed consent form in accordance with the Declaration of Helsinki. The informed consent form included a brief description about the study, as well as the confidentiality of their data in terms of remaining anonymous in any publication related to this study. It also informed them about their right to withdraw from the study at any time, and included contact information of the researchers so that participants could inquire about any further details of the study.

Participants were recruited via the Psychology School’s participant pool. Since web surveys are as effective as print surveys [40] and save more resources, a total of 92 Chinese adults (19 men and 73 women; *M*_age_ = 20.40 years, *SD* = 2.46) completed the experiment individually online through Sojump ([*https://www.wjx.cn/*)](https://www.wjx.cn/)), an online platform similar to Mechanical Turk. We prevented the submission of multiple responses from individual participants by restricting access from the same IP address in Experiments 1, 2, 3 and 4. All participants read the informed consent form and agreed to participate in the experiment. They received RMB 3 yuan via WeChat money transfer for their participation.

*(please see changes on page 8-9)*

Experiment 2

Participants were recruited via the Psychology School’s participant pool. A total of 97 Chinese adults (23 men and 74 women; *M*_age_ = 21.65 years, *SD* = 4.88) completed the experiment individually online through Sojump. All participants read the informed consent form and agreed to participate in the experiment. They received RMB 3 yuan via WeChat money transfer for their participation.

*(please see changes on page 13)*

Experiment 3

Participants were recruited via the Psychology School’s participant pool. A total of 60 Chinese adults (13 men and 47 women; *M*_age_ = 19.75 years, *SD* = 1.49) completed the experiment individually online through Sojump. All participants read the informed consent form and agreed to participate in the experiment. They received RMB 3 yuan via WeChat money transfer for their participation.

*(please see changes on page 16)*

Experiment 4

Participants were recruited via the Psychology School’s participant pool. A total of 150 Chinese adults (28 men and 122 women; *M*_age_ = 20.99 years, *SD* = 3.95) completed the experiment individually online through Sojump. All participants read the informed consent form and agreed to participate in the experiment. They received RMB 3 yuan via WeChat money transfer for their participation.

*(please see changes on page 19)*

*Sojump,* *an online platform similar to Mechanical Turk, is also known as Questionnaire Star or Wenjuanxing in Pinyin. To our knowledge, Sojump is currently the largest online data survey organization in China. Many researchers conduct questionnaires and behavioral experiments on it (Bai, B. Y., Liu, X. X., & Kou, Y., 2014; Klein, N., Grossmann, I., Uskul, A. K., Kraus, A. A., & Epley, N., 2015; Lien, C. H., & Cao, Y., 2014; Ruan, F., Fu, G., Zhou, M., Luo, L., Chen, J., Hua, W., at al., 2019; Yang, C., & Zhao, S., 2019; Zhang, J., Ziegler, M., & Paulhus, D. L., 2019). Since we recruited participants via the Psychology School’s participant pool, the respondents in our experiments were similar to convenience samples of students. Participants used the Sojump platform to complete the experiments on line. Moreover,* *the web surveys have the same effect as the print surveys (Huang, H., 2006). Therefore, we used this platform for data collection.*

**Third, the low numbers of respondents severely limit generalizability.**

*Response: Thank you for your comments. In the early version of the manuscript, the medium effect size is based on our unpublished manuscript on group potential preference. The effect size of that unpublished manuscript is similar to the effect size of potential preference in Tormala, Jia, & Norton’s (2012) study. In view of the fact that our study has not yet been published, we replaced the reference of the effect size of our unpublished manuscript with Tormala et al.’s (2012) study. In Tormala et al.’s study, the effect size is f=0.24. Under the conditions of α=.05 and a power of .90, the estimated sample size is the same as our actual sample size. The corresponding contents are as follows.*

Experiment 1

We used G*Power 3.1 [39] to calculate the desired sample size. We based our power analysis on previous research on the potential preference effects [6], which found an effect size of *f*= 0.24. To observe such an effect with an alpha of .05 and a power of .90, we needed a total sample of 92 participants.

*(please see changes on page 9)*

*In experiment 2, 3 and 4, we conducted a sensitivity power analysis respectively. The results showed that our sample sizes were sufficient to detect a medium effect size. Although our sample size may not be very large, we think that the sample sizes of the experiments are sufficient to detect a medium effect size. The corresponding contents are as follows.*

Experiment 2

We utilized a medium effect size of *f*=0.24, in line with past work on the potential preference effects [6] and Experiment 1. A sensitivity analysis conducted with G*Power [39] showed that our sample was sufficient to detect medium effects of *f*=0.26, assuming an alpha of .05 and a power of .90 for an ANOVA with repeated measures (observed correlation among repeated measures, *r*= -0.18).

*(please see changes on page 13)*

Experiment 3

Based on Experiments 1 and 2, we utilized a medium effect size of *f*=0.24. A sensitivity analysis conducted with G*Power [39] showed that our sample was sufficient to detect medium effects of *f*=0.24, assuming an alpha of .05 and a power of .90 for a within-participants ANOVA (observed correlation among repeated measures, *r*=0.22).

*(please see changes on page 16)*

Experiment 4

Based on Experiments 1, 2 and 3, we utilized a medium effect size of *f*=0.24. A sensitivity analysis conducted with G*Power [39] showed that our sample was sufficient to detect medium-to-large effects of *f*=0.29, assuming an alpha of .05 and a power of .90 for a one-way ANOVA.

*(please see changes on page 19).*

**Finally, the results of the study may in fact be caused by artifacts in the study design:**

**'moral improvement' was poorly operationalized, and respondents may have been more likely to give credence to reports that may be more salient (financial improvement and the potential for moral decline, as opposed to 'moral improvement' - do the authors know what that could consist of?)**

*Response: Thank you for your comments. We do often see reports of companies’ competence improvement and decline, as well as companies’ moral decline, in news and reports. We don’t often see news and reports on the companies’ moral improvement. We speculate that the lack of reports of the companies’ moral improvement may indicate that culturally, people may not attach importance to the companies’ moral improvement.*

*We believe that the companies’ moral improvement does exist and people can understand the companies’ moral improvement, because in the manipulation check, participants could well distinguish between moral improvement and stable morality. Moreover, if people can use certain criteria to judge the company’s moral decline, then they should also be able to judge the company’s moral improvement by the same criteria. Therefore, referring to the evaluation criteria of corporate morality of Ethisphere Institute* (see <https://ethisphere.com/128-worlds-most-ethical-companies-for-2019/>)*, we set up the company’s moral evaluation criteria as company ethics and compliance, company citizenship and responsibility, moral culture, company governance, and reputation. The decline in the company’s score in recent years indicates a decline in morality, while a rise in score indicates an improvement in morality.*

*However, we must admit that the hypothetical scenarios are not conducive to the generalization of research conclusions in reality. In the section of limitations and future directions, we discussed the impact of hypothetical scenarios on the generalization of research conclusions, as well as possible future research directions. The corresponding changes are as follows.*

We provided evidence for asymmetric biases regarding the potential for moral improvement and decline. However, before drawing strong conclusions, several limitations should be noted. First, to maintain overall logical consistency and internal validity, the scenarios we set are hypothetical. Although these scenarios ensure the internal validity of the experiment, extra caution is needed when generalizing the conclusions to a broader field. For example, the size of a group can affect people’s moral judgment of the group. Small groups are perceived to be more communal and trustworthy than big ones [50, 51]. Therefore, the moral potential of small companies may have a greater impact on an applicant’s willingness to join than for big companies. In addition, future research can explore the moderate effect of individual motivation on group potential preference. Applicants who want to change the future of the company may value the company’s moral and competence potential more than those who passively accept the status quo. Overall, testing the boundary conditions of asymmetric biases regarding a group’s moral potential and symmetrical biases with regards to its competence potential would facilitate more practical application of study findings.

*(please see changes on page 25).*

**Reviewer #4: In general, I believe this is an interesting project. The design seems adequate to test the hypotheses generated, though the methodology itself (particularly sample size) is of some concern. I have some concerns that I have outlined below:**

*Response: Thank you for your approval of research issue and research design.*

**Please provide more information regarding the recruitment platform. How are participants selected? Are participants fairly compensated? Can you confirm that participants in one study did not also participate in another?**

*Response: Sojump is an online platform similar to Mechanical Turk. To our knowledge, Sojump is currently the largest online data survey organization in China. Many researchers conduct questionnaires and behavioral experiments on it (Bai, B. Y., Liu, X. X., & Kou, Y., 2014; Klein, N., Grossmann, I., Uskul, A. K., Kraus, A. A., & Epley, N., 2015; Lien, C. H., & Cao, Y., 2014; Ruan, F., Fu, G., Zhou, M., Luo, L., Chen, J., Hua, W., at al., 2019; Yang, C., & Zhao, S., 2019; Zhang, J., Ziegler, M., & Paulhus, D. L., 2019). Participants were recruited via the Psychology School’s participant pool. We published the experimental information through the WeChat platform of the participant pool. When people read the experimental recruitment information and the corresponding remuneration and want to attend the experiment, they would use computers or mobile devices to participate by linking or scanning QR codes. Participants would receive a compensation of 3 yuan by WeChat transfer. We prevent repeated participation of the same person by restricting access from the same IP address, and the current experiments are limited to adults older than 18 years old in mainland China. To prevent the same participants from participating in different experiments, we reminded people not to participate repeatedly if they have attended the former experiments in the advertisements, otherwise they would not be paid accordingly. We set the filtering system so that users with the same IP address cannot participate in different experiments. The corresponding changes are as follows.*

Participants were recruited via the Psychology School’s participant pool. Since web surveys are as effective as print surveys [40] and save more resources, a total of 92 Chinese adults (19 men and 73 women; *M*_age_ = 20.40 years, *SD* = 2.46) completed the experiment individually online through Sojump ([*https://www.wjx.cn/*)](https://www.wjx.cn/)), an online platform similar to Mechanical Turk. We prevented the submission of multiple responses from individual participants by restricting access from the same IP address in Experiments 1, 2, 3 and 4. All participants read the informed consent form and agreed to participate in the experiment. They received RMB 3 yuan via WeChat money transfer for their participation.

*(please see changes on page 9)*

Experiment 2

Participants were recruited via the Psychology School’s participant pool. A total of 97 Chinese adults (23 men and 74 women; *M*_age_ = 21.65 years, *SD* = 4.88) completed the experiment individually online through Sojump. All participants read the informed consent form and agreed to participate in the experiment. They received RMB 3 yuan via WeChat money transfer for their participation.

*(please see changes on page 13)*

Experiment 3

Participants were recruited via the Psychology School’s participant pool. A total of 60 Chinese adults (13 men and 47 women; *M*_age_ = 19.75 years, *SD* = 1.49) completed the experiment individually online through Sojump. All participants read the informed consent form and agreed to participate in the experiment. They received RMB 3 yuan via WeChat money transfer for their participation.

*(please see changes on page 16)*

Experiment 4

Participants were recruited via the Psychology School’s participant pool. A total of 150 Chinese adults (28 men and 122 women; *M*_age_ = 20.99 years, *SD* = 3.95) completed the experiment individually online through Sojump. All participants read the informed consent form and agreed to participate in the experiment. They received RMB 3 yuan via WeChat money transfer for their participation.

*(please see changes on page 19)*

**Given the reliance of this work on experimental methods throughout, I would have liked a bit more details about the actual experiments. Particularly, although I understand that the study materials are not in english, english translations of the actual stimulus materials would be very much appreciated.**

*Response: We translate the experimental materials as follows.*

Experiment 1

Instruction 1 is the same in our four experiments, so we present Instruction 1 below, but not in the other three experiments’ materials to save space.

Instruction 1:

*Welcome to our research.*

*Center for Studies of Social Psychology, Central China Normal University supports the protection for human subjects involved in the study. The completion of this informed consent represents that you have reached the age of 18 and are willing to participate in the experiment. If you need to complain or have any other suggestions, please contact the researcher or the Human subjects Committee of Center for Studies of Social Psychology, CCNU.*

*Please check this item.*

*○I have read the consent form and voluntarily join this survey.*

Instruction 2:

*We are interested in how people evaluate and make decisions based on information about groups (such as companies and organizations). In this study, we will present information about two different groups. Please read the information and answer the following questions.*

Random: morality situation vs. competence situation

Morality situation: *A corporate morality evaluation institution evaluates the companies’ moral status in various industries every year. The assessment includes company ethics and compliance, company citizenship and responsibility, moral culture, company governance, and reputation.*

Competence situation: *A corporate competence evaluation institution evaluates the companies’ competence status in various industries every year. The assessment includes business performance, production and procurement ability, global marketing ability, international human resources, innovative research and development ability, and brand building ability.*

The following descriptions in morality situation is very similar to that in the competence situation, so we combine the two to save space.

*Company A and Company B are two companies with the same level of strength (morality) in the same industry.*

*The moral (competence) evaluation score of Company A has remained stable at about 95 in the past five years, and 95 in 2014 and 2018. The average score of moral (competence) evaluation of Company B is 89 in the past five years, 85 in 2014 and 93 in 2018, an average increase of about 2 points per year. The moral (competence) evaluation scores of Company A and Company B are shown in the following figure:*

*Morality (Competence) scores of Company A and Company B from 2014 to 2018.*

**A morality (competence) score of 95 means that the morality (competence) ranking is in the top 5% of the industry, and the score of 85 means it is in the top 15%.*

Questions:

*If you were an applicant, to what extent would you be willing to join Company A?*

*○not at all	○2	○3	○4	○5	○6	○very high*

*If you were an applicant, to what extent would you be willing to join Company B?*

*○not at all	○2	○3	○4	○5	○6	○very high*

*If you were an applicant, which company would you prefer to join?*

*○Company A	○Company B*

*Which company do you think has higher moral (competence) achievements?*

*○Company A	○Company B*

*Which company do you think has higher potential for moral (competence) improvement?*

*○Company A	○Company B*

Demographic information is collected at the end of each experiment, so we only present it here to save space.

Demographic information

*What is your gender?*

*○Male ○Female*

*What is your age?*

*(	)*

Experiment 2

Instruction 1: The same as Experiment 1.

Instruction 2:

*We are interested in how people evaluate and make decisions based on information about groups (such as companies and organizations). In this study, we will present information about two different groups. Please read the information and answer the following questions.*

Random: morality situation vs. competence situation

Morality situation: *A corporate morality evaluation institution evaluates the companies’ moral status in various industries every year. The assessment includes company ethics and compliance, company citizenship and responsibility, moral culture, company governance, and reputation.*

Competence situation: *A corporate competence evaluation institution evaluates the companies’ competence status in various industries every year. The assessment includes business performance, production and procurement ability, global marketing ability, international human resources, innovative research and development ability, and brand building ability.*

The following descriptions in morality situation is very similar to that in the competence situation, so we combine the two to save space.

*Company A and Company B are two companies with the same level of strength (morality) in the same industry.*

*The moral (competence) evaluation score of Company A has remained stable at about 85 in the past five years, and 85 in 2014 and 2018. The average score of moral (competence) evaluation of Company B was 91 in the past five years, 95 in 2014 and 87 in 2018, an average decrease of about 2 points per year. The moral (competence) evaluation scores of Company A and Company B are shown in the following figure:*

*Morality (Competence) scores of Company A and Company B from 2014 to 2018.*

**A morality (competence) score of 95 means that the morality (competence) ranking is in the top 5% of the industry, and the score of 85 means it is in the top 15%.*

Questions:

*If you were an applicant, to what extent would you be willing to join Company A?*

*○not at all	○2	○3	○4	○5	○6	○very high*

*If you were an applicant, to what extent would you be willing to join Company B?*

*○not at all	○2	○3	○4	○5	○6	○very high*

*If you were an applicant, which company would you prefer to join?*

*○Company A	○Company B*

*Which company do you think has higher moral (competence) achievements?*

*○Company A	○Company B*

*Which company do you think has higher potential for moral (competence) decline?*

*○Company A	○Company B*

Demographic information：the same as Experiment 1

Experiment 3

Instruction 1: The same as Experiment 1.

Instruction 2:

*We are interested in how people evaluate and make decisions based on information about groups (such as companies and organizations). In this study, we will present information about three different groups. Please read the information and answer the following questions.*

Random: potential for improvement vs. stable performance vs. potential for decline

*A corporate morality evaluation institution evaluates the companies’ moral status in various industries every year. The assessment includes company ethics and compliance, company citizenship and responsibility, moral culture, company governance, and reputation.*

*Company A, Company B and Company C are three companies with the same level of strength in the same industry.*

Potential for improvement: *The moral evaluation scores of Company A we*re *92.9 in 2016 and 95 in 2018. The average score of moral evaluation of Company A was 94 in the past three years.*

Stable performance: *The moral evaluation scores of Company B were 94.9 in 2016 and 95 in 2018. The average score of moral evaluation of Company B was 95 in the past three years.*

Potential for decline: *The moral evaluation scores of Company C were* 97*.1 in 2016 and 95 in 2018. The average score of moral evaluation of Company C was 96 in the past three years.*

*The moral evaluation scores of Company A, Company B and Company C are shown in the following figure:*

*Morality scores of Company A, Company B, and Company C from 2016 to 2018.*

**A morality score of 95 means that the morality ranking is in the top 5% of the industry, and the score of 96 means it is in the top 4%.*

Questions:

*If you were an applicant, to what extent would you be willing to join Company A?*

*○not at all	○2	○3	○4	○5	○6	○very high*

*If you were an applicant, to what extent would you be willing to join Company B?*

*○not at all	○2	○3	○4	○5	○6	○very high*

*If you were an applicant, to what extent would you be willing to join Company C?*

*○not at all	○2	○3	○4	○5	○6	○very high*

*If you were an applicant, which company would you prefer to join?*

*○Company A	○Company B	○Company C*

*Please rank the companies’ potential for moral improvement of Company A, Company B, and Company C, and rank the one you think has the highest potential as the first and the one with the lowest potential as the third.*

*○Company A	○Company B	○Company C*

*Please rank the companies’ potential for moral decline of Company A, Company B, and Company C, and rank the one you think has the highest potential as the first and the one with the lowest potential as the third.*

*○Company A	○Company B	○Company C*

Demographic information：the same as Experiment 1

Experiment 4

Instruction 1: The same as Experiment 1.

Instruction 2:

*We are interested in how people evaluate and make decisions based on information about groups (such as companies and organizations). In this study, we will present information about a group. Please read the information and answer the following questions.*

Random: stable performance vs. potential for improvement vs. potential for decline

*A corporate morality evaluation institution evaluates the companies’ moral status in various industries every year. The assessment includes company ethics and compliance, company citizenship and responsibility, moral culture, company governance, and reputation.*

Stable performance: *The moral evaluation scores of Company A were 95 in 2016 and 2018. The average score of moral evaluation of Company A was 95 in the past three years.*

Potential for improvement: *The moral evaluation scores of Company A were 94 in 2016 and 96 in 2018. The average score of moral evaluation of Company A was 95 in the past three years.*

Potential for decline: *The moral evaluation scores of Company A were 96 in 2016 and 94 in 2018. The average score of moral evaluation of Company A was 95 in the past three years.*

*The moral evaluation scores of Company A are shown in the following figure:*

*Morality scores of Company A from 2016 to 2018.*

**A morality score of 95 means that the morality ranking is in the top 5% of the industry, and the score of 96 means it is in the top 4%.*

Questions:

*If you were an applicant, to what extent would you be willing to join Company A?*

*○not at all	○2	○3	○4	○5	○6	○very high*

*What do you think is Company A’s potential for moral improvement?*

*○very low	○2	○3	○4	○5	○6	○very high*

Demographic information：the same as Experiment 1

**Throughout, the sample sizes are quite small. Although power analyses are reported in one part of the manuscript, they are not reported for all experiments. The size of samples leaves me concerned about the generalizability of the findings.**

*Response: Thank you for your comments. We adjusted the parameters to estimate the sample size of experiment 1. In the early version of manuscript, the medium effect size is based on our unpublished manuscript on group potential preference. The effect size of that unpublished manuscript is similar to the effect size of potential preference in Tormala, Jia, & Norton’s (2012) study. In view of the fact that our study has not yet been published, we replaced the reference of the effect size of our unpublished manuscript with Tormala et al.’s (2012) study. In Tormala et al.’s study, the effect size is f=0.24. Under the conditions of α=.05 and a power of .90, the estimated sample size is the same as our actual sample size. The corresponding changes are as follows.*

We used G*Power 3.1 [39] to calculate the desired sample size. We based our power analysis on previous research on the potential preference effects [6], which found an effect size of *f*= 0.24. To observe such an effect with an alpha of .05 and a power of .90, we needed a total sample of 92 participants.

*(please see changes on page 9).*

**Experiment 1:**

**Do not use terms like: "marginally significant." This is a meaningless phrase that relies on a statistic being close to an arbitrary cutoff. If your a-priori power analyses indicated that your alpha level was .05, anything that does not meet this is not significant. Please delete this language throughout.**

*Response: Thank you for your suggestion. We accept your suggestion and deleted the term of “marginally” in the manuscript. The corresponding changes are as follows.*

Although the effect of the interaction was not significant (*F*(1, 90) = 3.69, *p* = .058, *η*2 p = .04), we conducted a planned analysis, which was very important to test our hypothesis. Therefore, we analyzed simple main effects of dimension.

*(please see changes on page 11).*

**For Experiment 2, how was sample size determined?**

**For Experiment 3, how was sample size determined?**

**For Experiment 4, how was sample size determined?**

**In each of the above examples, is it assumed that the same power analyses from Experiment 1 still applied?**

*Response: Thank you for your comments. We think that the power analysis of Experiment 1 is not suitable for the other three experiments. Therefore, we conducted a sensitivity power analysis in experiment 2, 3 and 4 respectively. The results showed that our sample sizes were sufficient to detect a medium effect size. Although our sample sizes may not be very large, we think that the sample sizes of the experiments are sufficient to detect a medium effect size. The corresponding changes are as follows.*

Experiment 2

We utilized a medium effect size of *f*=0.24, in line with past work on the potential preference effects [6] and Experiment 1. A sensitivity analysis conducted with G*Power [39] showed that our sample was sufficient to detect medium effects of *f*=0.26, assuming an alpha of .05 and a power of .90 for an ANOVA with repeated measures (observed correlation among repeated measures, *r*= -0.18).

*(please see changes on page 13)*

Experiment 3

Based on Experiments 1 and 2, we utilized a medium effect size of *f*=0.24. A sensitivity analysis conducted with G*Power [39] showed that our sample was sufficient to detect medium effects of *f*=0.24, assuming an alpha of .05 and a power of .90 for a within-participants ANOVA (observed correlation among repeated measures, *r*=0.22).

*(please see changes on page 16)*

Experiment 4

Based on Experiments 1, 2 and 3, we utilized a medium effect size of *f*=0.24. A sensitivity analysis conducted with G*Power [39] showed that our sample was sufficient to detect medium-to-large effects of *f*=0.29, assuming an alpha of .05 and a power of .90 for a one-way ANOVA.

*(please see changes on page 19).*

**References**

*Bai, B. Y., Liu, X. X., & Kou, Y. (2014). Belief in a just world lowers perceived intention of corruption: the mediating role of perceived punishment. PLoS One, 9(5), e97075. doi:10.1371/journal.pone.0097075*

*Huang, H. (2006). Do print and Web surveys provide the same results? Computers in Human Behavior, 22(3), 334-350. doi:10.1016/j.chb.2004.09.012*

*Klein, N., Grossmann, I., Uskul, A. K., Kraus, A. A., & Epley, N. (2015). It pays to be nice, but not really nice: Asymmetric reputations from prosociality across 7 countries. Judgment & Decision Making, 10(4), 355-364.*

*Lien, C. H., & Cao, Y. (2014). Examining WeChat users’ motivations, trust, attitudes, and positive word-of-mouth: Evidence from China. Computers in Human Behavior, 41, 104-111. doi:10.1016/j.chb.2014.08.013*

*Ruan, F., Fu, G., Zhou, M., Luo, L., Chen, J., Hua, W., . . . Wang, J. (2019). Application of the Chinese version of Zelaya’s HIV-related stigma scale to undergraduates in mainland China. BMC Public Health, 19(1), 1708. doi:10.1186/s12889-019-8054-9*

*Tormala, Z. L., Jia, J. S., & Norton, M. I. (2012). The preference for potential. Journal of Personality and Social Psychology, 103(4), 567-583. doi:10.1037/a0029227*

*Yang, C., & Zhao, S. (2019). Determining preference for potential: The role of perceived economic mobility. Social Behavior and Personality: an international journal, 47(6), e8019. doi:10.2224/sbp.8019*

*Zhang, J., Ziegler, M., & Paulhus, D. L. (2019). Development and evaluation of the short Dark Triad – Chinese version (SD3-C). Current Psychology. doi:10.1007/s12144-019-00272-3*
