# Supplementary material for: The preference for potential in competence, not in morality: Asymmetric biases regarding a group's potential for moral improvement and decline
Source: PLoS One. 2020 Aug 4;15(8):e0236748. doi: 10.1371/journal.pone.0236748 (PMC7402473; doi:10.1371/journal.pone.0236748)
Supplement: Attachment — Submitted filename: Revision_20_05265.docx [file pone.0236748.s002.docx]

**Revision:** PONE-D-20-05265

The manuscript has improved considerably compared to the initial submission. It is good to see that the authors incorporated the literature suggested in the previous review rounds.

However, I have several more comments on this manuscript:

In experiment 2, the morality condition does not show significant results, p = .12, but is interpreted as if significant. This interpretation needs to be consistent across the paper, as insignificant results cannot be interpreted the same way as significant ones. The same problem appears in experiment 3 in the interpretation of the manipulation check, where p=.06 is interpreted as significant.

In all experiments, it’s unclear what the authors mean when they say that the same came from a psychology subject pool and was then conducted on an online platform similar to MTurk. Are all participants students who simply use the platform to receive their participation reward? If so, the analogy to MTurk is confusing, as people on MTurk are different in terms of demographics from students, and students would not be asked to use MTurk to complete studies.

On page 21, the first sentence of the results section is confusing. A significant effect of what? Of the experimental condition I assume, but this needs to be explicit.

The term asymmetry is not entirely clear, as it refers to the different effect of improvement vs. decline. I think this would be more clearly named non-linearity, or at the very least the asymmetry should be very clearly defined.

In the Discussion, I’m confused by the authors’ interpretation (p.24) that: “It seems that the morality of for-profit companies should neither improve nor decline. That situation creates a moral dilemma for for-profit companies.” Improvement on morality certainly won’t hurt, as their studies show, so why should a company not improve? Do they mean that given resource constraints, it might not be wise to invest in improvement?

More discussion of potential moderators of the asymmetry would be helpful. Would company size, entitativity, homogeneity influence the pattern? Would the effect differ depending on whether the morality is described as top-down actions from the company’s leadership versus part of a general company culture?
