# Supplementary material for: The preference for potential in competence, not in morality: Asymmetric biases regarding a group's potential for moral improvement and decline
Source: PLoS One. 2020 Aug 4;15(8):e0236748. doi: 10.1371/journal.pone.0236748 (PMC7402473; doi:10.1371/journal.pone.0236748)
Supplement: Attachment — Submitted filename: Response_to_Reviewers.docx [file pone.0236748.s003.docx]

*Dear Editor and Reviewers,*

*Thank you for your letter. We appreciate the reviewers’ approval of the revised contents and thank you for your new comments concerning our revised manuscript entitled “The preference for potential in competence, not in morality: Asymmetric biases regarding a group’s potential for moral improvement and decline” (ID: PONE-D-20-05265R1). These new comments are of great value to the improvement of our manuscript. We studied the comments carefully and have made corrections, hoping to meet your approval. The main corrections in the manuscript and the responds to the reviewers’ comments are as following:*

**Responds to the reviewers’ comments**

**Reviewer #1: Revision: PONE-D-20-05265**

**The manuscript has improved considerably compared to the initial submission. It is good to see that the authors incorporated the literature suggested in the previous review rounds.**

*Response: Thank you for your approval of the revised manuscript.*

**However, I have several more comments on this manuscript:**

**In experiment 2, the morality condition does not show significant results, p = .12, but is interpreted as if significant. This interpretation needs to be consistent across the paper, as insignificant results cannot be interpreted the same way as significant ones. The same problem appears in experiment 3 in the interpretation of the manipulation check, where p=.06 is interpreted as significant.**

*Response: Thank you for your comments. Although the result of the individual choice in the morality condition in experiment 2 and one of the results of the manipulation check in experiment 3 were consistent with our expectations, they did not reach statistical significance. To maintain the consistent of the interpretation of statistical significance, we revised the description of those two results. The corresponding changes in our manuscript are as follows.*

Similarly, in the morality condition, there was a tendency for more participants to select to join the stably moderate-performance company (30/49) than the high-performance company with high potential for decline (19/49; *χ*^2^(1) = 2.47, *p* = .12).

(please see changes on page 15)

Wilcoxon signed-rank tests showed that Company A (1.58) was ranked higher than Company B (2.07) (*Z* = 3.12, *p* < .01) and Company C (2.35) (*Z* = 3.02, *p* < .01), and Company B was ranked higher than Company C (*Z* = 1.89, *p* = .06), although this result did not reach statistical significance.

(please see changes on page 17)

**In all experiments, it’s unclear what the authors mean when they say that the sample came from a psychology subject pool and was then conducted on an online platform similar to MTurk. Are all participants students who simply use the platform to receive their participation reward? If so, the analogy to MTurk is confusing, as people on MTurk are different in terms of demographics from students, and students would not be asked to use MTurk to complete studies.**

*Response: Thank you for your comments. Our participants came from the Psychology School’s participant pool. We posted the recruitment information on the participants pool’s online platform, and the participants who were interested in the experiment participated the experiment through Sojump. Because there is a cooperative relationship between Sojump and WeChat, the participants who finished the experiments on Sojump were paid through WeChat transfer. If there are no college students in MTurk, then Mturk and Sojump are indeed different. Therefore, we delete that analogical statement from our manuscript. The corresponding changes in our manuscript are as follows.*

Because web surveys are as effective as print surveys [40] and save more resources, we chose to use the online survey platform Sojump ([*https://www.sojump.com*)](https://www.wjx.cn/)). A total of 92 Chinese adults (19 men and 73 women; *M*_age_ = 20.40 years, *SD* = 2.46) completed the experiment individually online.

*(please see changes on page 9)*

**On page 21, the first sentence of the results section is confusing. A significant effect of what? Of the experimental condition I assume, but this needs to be explicit.**

*Response: Thank you for your comments. We changed the statement of this result to make it clearer. The corresponding changes in our manuscript are as follows.*

An ANOVA revealed a significant effect of characteristic (*F*(2, 147) = 53.67, *p* < .001, *η*2 p = .42).

*(please see changes on page 21)*

**The term asymmetry is not entirely clear, as it refers to the different effect of improvement vs. decline. I think this would be more clearly named non-linearity, or at the very least the asymmetry should be very clearly defined.**

*Response: Thank you for your comments. To convey the meaning of the asymmetric biases more clearly, we defined this concept explicitly in the manuscript. The corresponding changes in our manuscript are as follows.*

Specifically, the asymmetric biases in this article are defined as a non-preference for the potential for moral improvement, but an aversion to the potential for moral decline.

*(please see changes on page 5-6)*

**In the Discussion, I’m confused by the authors’ interpretation (p.24) that: “It seems that the morality of for-profit companies should neither improve nor decline. That situation creates a moral dilemma for for-profit companies.” Improvement on morality certainly won’t hurt, as their studies show, so why should a company not improve? Do they mean that given resource constraints, it might not be wise to invest in improvement?**

*Response: Thank you for your comments. The statement of “the morality of for-profit companies should neither improve nor decline” is not very clear. What we are trying to convey is that the moral improvement of for-profit companies may lead to a decline in people’s evaluation of their competence, so for-profit companies’ moral improvement may have negative effects. To make it clearer, we changed the corresponding contents. The corresponding changes in our manuscript are as follows.*

The situation is a little more subtle for the morality of for-profit companies. Although for-profit companies have made important contributions to social progress, they are stereotypically depicted as immoral and harmful to society [44]. On the contrary, the moral improvement of for-profit companies is considered to hinder their competence [34]. It seems that for-profit companies should pay more attention to the development of their competence. However, no matter how competitive a for-profit company is, any moral decline or scandal may put the company in jeopardy [36-38]. To sum up, the moral improvement of for-profit companies may lead to a decline in people’s evaluation of the companies’ competence, while the companies’ moral decline may lead to people’s worse overall impressions of them. It seems that both the moral improvement and decline of for-profit companies may have negative effects. That situation creates a moral dilemma for for-profit companies. Future research could further test the effect of for-profit corporate moral dilemma.

*(please see changes on page 24)*

**More discussion of potential moderators of the asymmetry would be helpful. Would company size, entitativity, homogeneity influence the pattern? Would the effect differ depending on whether the morality is described as top-down actions from the company’s leadership versus part of a general company culture?**

*Response: Thank you for your insightful suggestions. According to your suggestion in the previous review, we discussed the influence of group size on the asymmetric biases in the previous revision. Following your suggestion in the current review, we found that the entitativity could also affect people’s evaluation of group morality. Therefore, we discussed why the entitativity might also be a potential moderator of the asymmetry. Moreover, the characteristics of group morality and the individual motivation may also have an impact on the asymmetry. We discussed those issues in the manuscript. The corresponding changes in our manuscript are as follows.*

For example, the size and the entitativity of a group can affect people’s moral judgment of the group. Small groups are perceived to be more communal and trustworthy than big ones [51, 52]. Therefore, the moral potential of small companies may have a greater impact than big companies on an applicant’s willingness to join. Perceived entitativity could make people’s moral judgment of the group more extreme. People are more averse to the immoral behaviors of the high entitativity group than the low entitativity group, but they prefer the moral behaviors of the high entitativity group [53, 54]. Therefore, group entitativity may moderate people’s attitude towards the group’s moral improvement and decline. In addition, future research can explore the moderating effect of the characteristics of a company’s morality and the individual motivation on the asymmetric biases regarding a group’s potential for moral improvement and decline. The company’s moral improvement and decline may be caused by the corporate culture or the top-down actions of the company’s leadership. Because the corporate culture is relatively stable, people may deem that a company’s moral improvement and decline driven by leadership is not as lasting as the changes brought about by the corporate culture. Therefore, the corporate culture may have a greater impact on people’s attitude towards a company’s moral improvement and decline than the company’s leadership does. Moreover, applicants who want to change the future of the company may value the company’s moral potential more than those who passively accept the status quo. Overall, testing the boundary conditions of asymmetric biases regarding a group’s moral potential would facilitate more practical application of study findings.

*(please see changes on page 25-26)*

**Reviewer #2: One last comment: Regarding the sentence "the improvement and decline of morality may correspond to different dimensions, such as prescriptive and proscriptive morality, respectively", I found it reluctant to categorize moral improvement/decline into prescriptive/proscriptive morality as "two moral dimensions". Dimensions are usually descriptive of different contents but not the trend of the same content. I listed this reference earlier, but may be misinterpreted by the authors. A better way to put it may be the "bad is stronger than good" effect. The authors may think how to better structure the relevance of the theories and their findings.**

**Baumeister, R. F., Bratslavsky, E., Finkenauer, C., & Vohs, K. D. (2001). Bad is stronger than good. Review of General Psychology, 5(4), 323-370.**

*Response: Thank you for your comments. It is indeed inappropriate to directly associate moral improvement and decline with prescriptive and proscriptive morality, respectively. We proposed that people might associate moral improvement and decline with moral and immoral behaviors, respectively. It may be that people’s asymmetric attitudes towards the moral and immoral behaviors lead to people’s asymmetric biased regarding the moral potential. However, we think that the “bad is stronger than good” effect may also have an impact on the asymmetric biases regarding the moral potential. The corresponding changes in our manuscript are as follows.*

However, the asymmetric biases regarding the potential for moral improvement and decline indicate that people may process the information of moral improvement and decline differently. Although we manipulated the potential for moral improvement and decline in a similar manner, people may have associated moral improvement and decline with moral and immoral behaviors, respectively. Research shows that people do not force others to become more moral, but they cannot tolerate others becoming more immoral [29]. Consequently, people who perform different degrees of good deeds may be perceived to have the same degree of kindness, but the more bad things they do, the worse they are [31, 32]. In addition, bad actions are often more powerful than good ones [43]. Therefore, although people have no preference for the potential for moral improvement, they are averse to the potential for moral decline.

*(please see changes on page 23)*
