# Supplementary material for: The preference for potential in competence, not in morality: Asymmetric biases regarding a group's potential for moral improvement and decline
Source: PLoS One. 2020 Aug 4;15(8):e0236748. doi: 10.1371/journal.pone.0236748 (PMC7402473; doi:10.1371/journal.pone.0236748)
Supplement: Peer review history

# Peer review: Decision Letter 0

**DOI:** 10.1371/journal.pone.0236748.r001

**Authors:** Baogui Xin

13 Apr 2020

PONE-D-20-05265

The preference for potential in competence, not in morality: Asymmetric biases regarding a group's potential for moral improvement and decline

PLOS ONE

Dear Mr Zuo,

Thank you for submitting your manuscript to PLOS ONE. After careful consideration, we feel that it has merit but does not fully meet PLOS ONE’s publication criteria as it currently stands. Therefore, we invite you to submit a revised version of the manuscript that addresses the points raised during the review process.

Though half of reviewers suggested us to reject PONE-D-20-05265, all reviewers provided many valuable and constructive comments. Considering reviewers’ useful comments and the interesting topic of the manuscript, I would like to give you a chance to revise your manuscript during the special period. The revised manuscript will undergo the next round of review by the same reviewers.

We would appreciate receiving your revised manuscript by May 28 2020 11:59PM. When you are ready to submit your revision, log on to https://www.editorialmanager.com/pone/ and select the 'Submissions Needing Revision' folder to locate your manuscript file.

If you would like to make changes to your financial disclosure, please include your updated statement in your cover letter.

To enhance the reproducibility of your results, we recommend that if applicable you deposit your laboratory protocols in protocols.io, where a protocol can be assigned its own identifier (DOI) such that it can be cited independently in the future. For instructions see: http://journals.plos.org/plosone/s/submission-guidelines#loc-laboratory-protocols

Please include the following items when submitting your revised manuscript:

- A rebuttal letter that responds to each point raised by the academic editor and reviewer(s). This letter should be uploaded as separate file and labeled 'Response to Reviewers'.
- A marked-up copy of your manuscript that highlights changes made to the original version. This file should be uploaded as separate file and labeled 'Revised Manuscript with Track Changes'.
- An unmarked version of your revised paper without tracked changes. This file should be uploaded as separate file and labeled 'Manuscript'.

Please note while forming your response, if your article is accepted, you may have the opportunity to make the peer review history publicly available. The record will include editor decision letters (with reviews) and your responses to reviewer comments. If eligible, we will contact you to opt in or out.

We look forward to receiving your revised manuscript.

Kind regards,

Baogui Xin, Ph.D.

Academic Editor

PLOS ONE

Journal Requirements:

When submitting your revision, we need you to address these additional requirements.

1. Please ensure that your manuscript meets PLOS ONE's style requirements, including those for file naming. The PLOS ONE style templates can be found at

http://www.journals.plos.org/plosone/s/file?id=wjVg/PLOSOne_formatting_sample_main_body.pdf and http://www.journals.plos.org/plosone/s/file?id=ba62/PLOSOne_formatting_sample_title_authors_affiliations.pdf

2. Please include additional information regarding the survey or questionnaire used in the study and ensure that you have provided sufficient details that others could replicate the analyses. For instance, if you developed a questionnaire as part of this study and it is not under a copyright more restrictive than CC-BY, please include a copy, in both the original language and English, as Supporting Information.

3. In the ethics statement in the Methods and online submission information, please clarify whether you obtained written informed consent from the participants (or whether participation implied consent). If the need for consent was waived by the ethics committee, please include this information.

[Note: HTML markup is below. Please do not edit.]

Reviewers' comments:

Reviewer's Responses to Questions

**Comments to the Author**

1. Is the manuscript technically sound, and do the data support the conclusions?

The manuscript must describe a technically sound piece of scientific research with data that supports the conclusions. Experiments must have been conducted rigorously, with appropriate controls, replication, and sample sizes. The conclusions must be drawn appropriately based on the data presented. 

Reviewer #1: Yes

Reviewer #2: Yes

Reviewer #3: Partly

Reviewer #4: Partly

**********

2. Has the statistical analysis been performed appropriately and rigorously? 

Reviewer #1: Yes

Reviewer #2: Yes

Reviewer #3: Yes

Reviewer #4: Yes

**********

3. Have the authors made all data underlying the findings in their manuscript fully available?

The PLOS Data policy requires authors to make all data underlying the findings described in their manuscript fully available without restriction, with rare exception (please refer to the Data Availability Statement in the manuscript PDF file). The data should be provided as part of the manuscript or its supporting information, or deposited to a public repository. For example, in addition to summary statistics, the data points behind means, medians and variance measures should be available. If there are restrictions on publicly sharing data—e.g. participant privacy or use of data from a third party—those must be specified.

Reviewer #1: Yes

Reviewer #2: Yes

Reviewer #3: No

Reviewer #4: Yes

**********

4. Is the manuscript presented in an intelligible fashion and written in standard English?

PLOS ONE does not copyedit accepted manuscripts, so the language in submitted articles must be clear, correct, and unambiguous. Any typographical or grammatical errors should be corrected at revision, so please note any specific errors here.

Reviewer #1: Yes

Reviewer #2: Yes

Reviewer #3: No

Reviewer #4: Yes

**********

5. Review Comments to the Author

Please use the space provided to explain your answers to the questions above. You may also include additional comments for the author, including concerns about dual publication, research ethics, or publication ethics. (Please upload your review as an attachment if it exceeds 20,000 characters)

Reviewer #1: Review PONE-D-20-05265

The authors test whether people preferred groups depending on whether their morality versus competence was expected to improve versus decline. The question is interesting per se, and the authors offers 4 experiments that test this question.

I see several avenues for improvement of the manuscript:

In the opening example, it would be good to be a bit more concrete. How would someone know that a company is expected to decline in morality in the real world, what would indicate that?

The authors should make clearer whether they expect the same patterns found for the morality and competence of individuals to also hold for the qualities of organizations. This assumption is now somewhat implicit in the theory part, but it should be made clearer by flagging which cited literature pertains to individuals versus organizations in the theoretical introduction. See Critcher & Dunning 2014 or Skowronski 2002 for differences and similarities in the perception of groups versus individuals.

The authors should mention in the abstract that they conducted 4 studies.

In the concrete experiments, the scenarios are very hypothetical. It’s not clear what size the companies would have, in which role the participants imagined to join, and whether they’d imagine any potential to change the company’s morality versus competence. This assessment would probably be different whether someone expects to join a 5-people start-up versus a large company in a minor role. See for example La Macchia, Louis, Hornsey & Leonardelli 2016 on relevant differences between the morality of small and large groups. The authors should discuss or test whether small versus large groups would be some boundary condition.

The terminology is inconsistent at times. In the Pilot study, the authors talk about warmth instead of morality, but these are not the same thing. This needs to be clarified or the study needs to be replaced.

The mechanism underlying the asymmetry between morality and competence should be explained better. Is it that for morality, there is some sort of threshold, where once this is reached, more is not better? In contrast, for competence, more is always better? This should at the very least be elaborated on in the Discussion. The section on Asymmetry is quire repetitive, it would be good to raise some more substantial points here.

Experiment 3 seems somewhat redundant. It would be nice to show a behavioral consequence of the effect, eg willingness to join groups when the outcome is less hypothetical and more personally relevant. Alternatively, participants could be asked to bet some small amount on the group. At the very least, it should be discussed in the limitations in light of existing work that shows similar effects for hypothetical versus costly behavior in performance vs. morality perception (Cone & Ferguson, 2015; Steinmetz, Toure-Tillery, & Fishbach, 2020).

Reviewer #2: The manuscript was generally well-written. The authors presented four rigorous experiments, illustrating people’s (as)symmetric preference for moral-/competence-characterized groups. The line of reasoning was comprehensible, and the experiments were clearly communicated.

Despite so, a few remaining questions can be better addressed, specifically as below:

1. In the authors’ reasoning about why people demonstrate asymmetric biases in moral improvement versus decline, a core concept “insensitivity” emerged. What does “insensitivity” mean? People are less capable to distinguish? OR less motivated to react to the stimuli? The exact implication of this phrase is somehow vague, and is not fully convincing when referring to the data. For example:

1a. In reasoning of “insensitivity”, on page 5, the first paragraph presented empirical evidence on how people are less likely to distinguish morally good deeds than morally bad deeds, while the second paragraph demonstrated studies showing that people are less likely to rely on morally positive than negative information to determine their overall impression. In at least these two examples, “insensitivity” implies different things.

1b. When looking at the actual data, people can perfectly distinguish which companies are improving, stable, and declining (according to the manipulation check). So, it seems to me that people just care less about morality in its upward than downward trend.

Concerning the critical role of “insensitivity” in the line of reasoning, this concept deserves better clarification, or replacement with more appropriate expressions (valuation, weight, etc.).

2. The focal research question (as presented in the DV, “If you were an applicant, to what extent would you be willing to join Company …?”) focuses on people’s intended interaction with group (especially business-framed group emphasizing competence) rather than individual. However, most of the literature overemphasized stereotype content influences on individual perception and interaction. It may be plausible to posit that, for example, people are less willing to join morally improving companies since moral improvement of companies can compromise its competence (Aaker, Vohs, & Mogilner, 2010) and efficiencies in promoting applicants’ self-benefits. And morally positive targets can be perceived as less moral when they threaten self-interest (Melnikoff & Bailey, 2018). OR, negative moral changes usually stimulate more sanctions than positive ones (Janoff-Bulman, Sheikh, & Hepp, 2009), which also pose higher influence on potential applicants’ self-interest.

2a. Thus, I expect that the authors can provide more reasoning and literature regarding why people interact with companies and organizations (especially in business-oriented mindset or situations; rather than individuals) depending on their moral-related stereotype contents.

2b. Relatedly, page 5 line 112, it seems irrelevant to discuss morality and identity judgment. Self-concept and other-perception are different domains and can have different cognitive mechanisms.

3. Two minor issues:

3a. Sample size across the studies should be sufficient; however, design in Study 4 (between-subjects) was quite different from previous ones (within-subjects). At least, a sensitivity power analysis should be conducted to justify the sample of Study 4.

3b. On page 22, why did the authors only examine potential for moral improvement in the manipulation check? The missing check of moral decline is not equivalent to that in previous studies.

References

Aaker, J., Vohs, K. D., & Mogilner, C. (2010). Nonprofits are seen as warm and for-profits as competent: Firm stereotypes matter. Journal of Consumer Research, 37(2), 224-237.

Janoff-Bulman, R., Sheikh, S., & Hepp, S. (2009). Proscriptive versus prescriptive morality: Two faces of moral regulation. Journal of personality and social psychology, 96(3), 521.

Melnikoff, D. E., & Bailey, A. H. (2018). Preferences for moral vs. immoral traits in others are conditional. Proceedings of the National Academy of Sciences, 115(4), E592-E600.

Reviewer #3: There are many issues with this paper.

First and foremost, equating 'warmth' with 'morality' is problematic, and the paper jumps from intent in the introduction to 'warmth' to 'strength (ethics)' to 'morality (competence)'. This is very confusing as it seems that the authors keep switching what they are writing about and are mixing very different things.

Second, the paper does not provide crucial information regarding the recruitment methods:

were there incentives given to participants?

did they complete informed consent?

what efforts were taken to ensure participant privacy and data protection?

what is the literature on the characteristics and reliability of Sojump respondents (e.g., compared to convenience samples of students or to representative samples)?

Third, the low numbers of respondents severely limit generalizability.

Finally, the results of the study may in fact be caused by artifacts in the study design:

'moral improvement' was poorly operationalized, and respondents may have been more likely to give credence to reports that may be more salient (financial improvement and the potential for moral decline, as opposed to 'moral improvement' - do the authors know what that could consist of?)

Reviewer #4: In general, I believe this is an interesting project. The design seems adequate to test the hypotheses generated, though the methodology itself (particularly sample size) is of some concern. I have some concerns that I have outlined below:

Please provide more information regarding the recruitment platform. How are participants selected? Are participants fairly compensated? Can you confirm that participants in one study did not also participate in another?

Given the reliance of this work on experimental methods throughout, I would have liked a bit more details about the actual experiments. Particularly, although I understand that the study materials are not in english, english translations of the actual stimulus materials would be very much appreciated.

Throughout, the sample sizes are quite small. Although power analyses are reported in one part of the manuscript, they are not reported for all experiments. The size of samples leaves me concerned about the generalizability of the findings.

Experiment 1:

Do not use terms like: "marginally significant." This is a meaningless phrase that relies on a statistic being close to an arbitrary cutoff. If your a-priori power analyses indicated that your alpha level was .05, anything that does not meet this is not significant. Please delete this language throughout.

For Experiment 2, how was sample size determined?

For Experiment 3, how was sample size determined?

For Experiment 4, how was sample size determined?

In each of the above examples, is it assumed that the same power analyses from Experiment 1 still applied?

**********

6. PLOS authors have the option to publish the peer review history of their article (what does this mean?). If published, this will include your full peer review and any attached files.

If you choose “no”, your identity will remain anonymous but your review may still be made public.

**Do you want your identity to be public for this peer review?** For information about this choice, including consent withdrawal, please see our Privacy Policy.

Reviewer #1: No

Reviewer #2: No

Reviewer #3: No

Reviewer #4: No

[NOTE: If reviewer comments were submitted as an attachment file, they will be attached to this email and accessible via the submission site. Please log into your account, locate the manuscript record, and check for the action link "View Attachments". If this link does not appear, there are no attachment files to be viewed.]

While revising your submission, please upload your figure files to the Preflight Analysis and Conversion Engine (PACE) digital diagnostic tool, https://pacev2.apexcovantage.com/. PACE helps ensure that figures meet PLOS requirements. To use PACE, you must first register as a user. Registration is free. Then, login and navigate to the UPLOAD tab, where you will find detailed instructions on how to use the tool. If you encounter any issues or have any questions when using PACE, please email us at figures@plos.org. Please note that Supporting Information files do not need this step.

# Author comment: Author response to Decision Letter 0

**DOI:** 10.1371/journal.pone.0236748.r002

31 May 2020

Responds to the reviewers’ comments

Reviewer #1: Review PONE-D-20-05265

The authors test whether people preferred groups depending on whether their morality versus competence was expected to improve versus decline. The question is interesting per se, and the authors offers 4 experiments that test this question.

Response: Thank you for your approval of the research issue.

I see several avenues for improvement of the manuscript:

In the opening example, it would be good to be a bit more concrete. How would someone know that a company is expected to decline in morality in the real world, what would indicate that?

Response: Thank you for your comments. In the real world, people can learn about a company’s morality situation by checking the assessment released by rating agencies. For example, Ethisphere Institute rates the most ethical companies in the world every recent year. If a company’s moral ranking has declined linearly in recent years, people may predict that the company’s morality would continue to decline in the future. 

The authors should make clearer whether they expect the same patterns found for the morality and competence of individuals to also hold for the qualities of organizations. This assumption is now somewhat implicit in the theory part, but it should be made clearer by flagging which cited literature pertains to individuals versus organizations in the theoretical introduction. See Critcher & Dunning 2014 or Skowronski 2002 for differences and similarities in the perception of groups versus individuals.

Response: Thank you for your comments. In the part of the theoretical review of the symmetric biases regarding competence potential, we explained the similarities and differences in people’s processing of individual and group information. In some ways, people’s perception of the individual and the group is different. For example, compared with the group, people judge the individual more positively. However, in the face of linear development trend, people’s expectations for the future of individuals and groups are similar. In addition, people give more value to future achievements than current achievements. Therefore, people may prefer the individual’s and group’s potential for improvement in terms of competence, and have an aversion to their corresponding potential for decline. We clearly pointed out that the symmetric biases regarding the potential of individual’s and group’s competence both exist. 

In the part of the theoretical review of the asymmetric biases regarding moral potential, following he the suggestions of Reviewer #2, we deleted the literature review on identity judgment and replaced it with literature review on how people perceive group morality. 

The authors should mention in the abstract that they conducted 4 studies.

Response: Thank you for your suggestion. In the abstract, we make it clear that 4 experiments have been done in the research. 

In the concrete experiments, the scenarios are very hypothetical. It’s not clear what size the companies would have, in which role the participants imagined to join, and whether they’d imagine any potential to change the company’s morality versus competence. This assessment would probably be different whether someone expects to join a 5-people start-up versus a large company in a minor role. See for example La Macchia, Louis, Hornsey & Leonardelli 2016 on relevant differences between the morality of small and large groups. The authors should discuss or test whether small versus large groups would be some boundary condition.

Response: Thank you for your comments. In the discussion section, we discussed how the hypothetical scenarios affect the internal and external validity of the research. Although the hypothetical scenarios are conducive to the internal consistency of the whole research logic, it is not conducive to the generalization of conclusions and the exploration of boundary conditions. We discussed the possible moderating effects of company size and applicants’ motivation on the asymmetric biases regarding groups’ moral potential. 

The terminology is inconsistent at times. In the Pilot study, the authors talk about warmth instead of morality, but these are not the same thing. This needs to be clarified or the study needs to be replaced.

Response: Thank you for your comments. Warmth assessed in the pilot experiment is indeed inconsistent with the terminology of the whole study. In view of the fact that the deletion of the pilot experiment has no effect on the logic and the integrity of the results of the whole study, we decided to delete the pilot experiment to maintain the consistency of the research terminology and logic.

The mechanism underlying the asymmetry between morality and competence should be explained better. Is it that for morality, there is some sort of threshold, where once this is reached, more is not better? In contrast, for competence, more is always better? This should at the very least be elaborated on in the Discussion. The section on Asymmetry is quire repetitive, it would be good to raise some more substantial points here.

Response: Thank you for your comments. The section on asymmetry really does not give a more in-depth explanation of the mechanism behind asymmetry, so we rewrote all the contents of this section. As you said, the reason why moral improvement does not lead to higher evaluation may be that there is a sort of threshold in the evaluation of morality. Specifically, the threshold may divide morality into two dimensions: prescriptive morality and proscriptive morality. Prescriptive morality is sensitive to positive outcomes, based on activation, and focuses on what we should do. Whereas proscriptive morality is sensitive to negative outcomes, based on inhibition, and focuses on what we should not do. The improvement of prescriptive morality may not lead to higher evaluation, while the decline of proscriptive morality may lead to lower evaluation. Therefore, people do not prefer the potential for moral improvement, but are averse to the potential for moral decline.

However, there may be no such threshold in the evaluation of competence. The change of competence could be perceived as change in one dimension. The improvement of competence leads to higher evaluation, while the decline of competence leads to lower evaluation. 

We also discussed the possible mechanism behind people’s asymmetric biases regarding the moral potential of for-profit companies. 

Experiment 3 seems somewhat redundant. It would be nice to show a behavioral consequence of the effect, eg willingness to join groups when the outcome is less hypothetical and more personally relevant. Alternatively, participants could be asked to bet some small amount on the group. At the very least, it should be discussed in the limitations in light of existing work that shows similar effects for hypothetical versus costly behavior in performance vs. morality perception (Cone & Ferguson, 2015; Steinmetz, Toure-Tillery, & Fishbach, 2020).

Response: Thank you for your comments. Based on our current resources and limited time, we are sorry that we may not be able to complete the new experiment. But your suggestion is very instructive to the future research. In the section of limitations and future directions, we discussed the influence of the diversity of dependent variables on the internal and external validity of the study. 

On the one hand, according to the literature you provided (Cone & Ferguson, 2015; Steinmetz, Toure-Tillery, & Fishbach, 2020), the hypothetical decision is consistent with the decision that needs to pay the price. People’s hypothetical decisions in situations where there is no need to pay a price could be extended to costly behaviors. On the other hand, research shows that hypothetical decisions are different from real-life decisions. Moreover, the decision-making situation could moderate the potential preference effect. Specifically, in abstract and hedonic decision-making situations, people prefer potential to achievements, while in concrete and utilitarian decision-making situations, people prefer achievements to potential. It is not clear whether the decision-making situation could moderate the asymmetric biases regarding group’s moral potential. This issue is very interesting and could be explored more deeply in future research. 

Reviewer #2: The manuscript was generally well-written. The authors presented four rigorous experiments, illustrating people’s (as)symmetric preference for moral-/competence-characterized groups. The line of reasoning was comprehensible, and the experiments were clearly communicated.

Response: Thank you for your approval of the research problem and the whole research logic.

Despite so, a few remaining questions can be better addressed, specifically as below:

1. In the authors’ reasoning about why people demonstrate asymmetric biases in moral improvement versus decline, a core concept “insensitivity” emerged. What does “insensitivity” mean? People are less capable to distinguish? OR less motivated to react to the stimuli? The exact implication of this phrase is somehow vague, and is not fully convincing when referring to the data. For example:

1a. In reasoning of “insensitivity”, on page 5, the first paragraph presented empirical evidence on how people are less likely to distinguish morally good deeds than morally bad deeds, while the second paragraph demonstrated studies showing that people are less likely to rely on morally positive than negative information to determine their overall impression. In at least these two examples, “insensitivity” implies different things.

1b. When looking at the actual data, people can perfectly distinguish which companies are improving, stable, and declining (according to the manipulation check). So, it seems to me that people just care less about morality in its upward than downward trend.

Concerning the critical role of “insensitivity” in the line of reasoning, this concept deserves better clarification, or replacement with more appropriate expressions (valuation, weight, etc.).

Response: Thank you for your comments. As you said, the concept of “insensitivity” doesn’t explain the asymmetric biases very well. In the experimental manipulation, people can distinguish between the improvement and decline of group morality in different degrees. They just don’t seem to care too much about the improvement of group morality. In view of the references you have provided (Janoff-Bulman, Sheikh & Hepp, 2009), the improvement and decline of group morality are likely to correspond to prescriptive and proscriptive morality, respectively. People do not have mandatory requirements for prescriptive morality, so different degrees of moral improvement are acceptable. However, people can’t accept the violation of proscriptive morality. Therefore, following your suggestion, we changed the term “insensitivity” to “evaluation”. That is, the evaluations of the improvement and decline of morality are asymmetric.

We also discussed this issue in the discussion section. The symmetric biases regarding competence potential may be due to that the improvement and decline of competence are in one dimension, whereas the asymmetric biases regarding moral potential may be due to that the improvement and decline of morality correspond to two dimensions of morality respectively. It may be that the two dimensions of morality have different attributes that lead to people’s asymmetric biases regarding the potential of moral improvement and decline. 

2. The focal research question (as presented in the DV, “If you were an applicant, to what extent would you be willing to join Company …?”) focuses on people’s intended interaction with group (especially business-framed group emphasizing competence) rather than individual. However, most of the literature overemphasized stereotype content influences on individual perception and interaction. It may be plausible to posit that, for example, people are less willing to join morally improving companies since moral improvement of companies can compromise its competence (Aaker, Vohs, & Mogilner, 2010) and efficiencies in promoting applicants’ self-benefits. And morally positive targets can be perceived as less moral when they threaten self-interest (Melnikoff & Bailey, 2018). OR, negative moral changes usually stimulate more sanctions than positive ones (Janoff-Bulman, Sheikh, & Hepp, 2009), which also pose higher influence on potential applicants’ self-interest.

2a. Thus, I expect that the authors can provide more reasoning and literature regarding why people interact with companies and organizations (especially in business-oriented mindset or situations; rather than individuals) depending on their moral-related stereotype contents.

2b. Relatedly, page 5 line 112, it seems irrelevant to discuss morality and identity judgment. Self-concept and other-perception are different domains and can have different cognitive mechanisms.

Response: Thank you for your insightful ideas on this study. We reviewed the literature on the evaluations of individuals to companies, and proposed that there may be a dilemma in people’s attitudes towards for-profit companies’ morality. The purpose of a for-profit company is to make bigger profits, and people may join the job to get a higher salary. To this end, for-profit companies need to constantly improve their competence. However, the improvement of corporate morality is likely to reduce people’s evaluation of the company’s competence, thus affecting the interests of its employees. As potential employees, applicants are likely to lower their evaluation of the company’s moral improvement. Meanwhile, the company’s moral decline would undoubtedly lower people’s evaluation of the company. Therefore, there may be a moral dilemma for for-profit companies. 

We agree with you that the relationship between identity judgment and the purpose of this study is weak. Although this line of research on identity judgment supports people’s emphasis on the moral decline in judging other’s identity, it focuses on the cognition of identity judgment and has a weak relationship with the perception of group value. Therefore, we deleted the literature review and the related discussions about identity judgment in the manuscript.

3. Two minor issues:

3a. Sample size across the studies should be sufficient; however, design in Study 4 (between-subjects) was quite different from previous ones (within-subjects). At least, a sensitivity power analysis should be conducted to justify the sample of Study 4.

Response: Thank you for your approval of the sample size of the experiments. We have modified the calculation parameters and reference of effect size of a priori analysis in experiment 1. 

In experiment 2, 3 and 4, we conducted a sensitivity power analysis, which shows that the sample sizes are sufficient to detect medium effect sizes. 

3b. On page 22, why did the authors only examine potential for moral improvement in the manipulation check? The missing check of moral decline is not equivalent to that in previous studies.

Response: Thank you for your comments. In experiment 4, we only set up the manipulation check on the potential for moral improvement, but did not test the manipulation on the potential for moral decline, which is really different from experiment 3. We did this because in experiment 4, we mainly wanted to test whether people still have no preference for the potential for moral improvement in more extreme situations. 

In this situation, the average moral levels of the three companies are the same, and in the last year, the moral level of the company with potential for improvement has even exceeded that of the company with stable morality. However, the dual advantages of the company with potential for moral improvement (high morality in the recent year and high potential for moral improvement) have not made people prefer it. 

In this situation, however, the company with potential for moral decline have double disadvantages (low morality in the recent year and high potential for moral decline). In this case, people would undoubtedly be more averse to the company with potential for moral decline. Therefore, we only set up manipulation check on the potential for moral improvement.

References

Aaker, J., Vohs, K. D., & Mogilner, C. (2010). Nonprofits are seen as warm and for-profits as competent: Firm stereotypes matter. Journal of Consumer Research, 37(2), 224-237.

Janoff-Bulman, R., Sheikh, S., & Hepp, S. (2009). Proscriptive versus prescriptive morality: Two faces of moral regulation. Journal of personality and social psychology, 96(3), 521.

Melnikoff, D. E., & Bailey, A. H. (2018). Preferences for moral vs. immoral traits in others are conditional. Proceedings of the National Academy of Sciences, 115(4), E592-E600.

Reviewer #3: There are many issues with this paper.

First and foremost, equating 'warmth' with 'morality' is problematic, and the paper jumps from intent in the introduction to 'warmth' to 'strength (ethics)' to 'morality (competence)'. This is very confusing as it seems that the authors keep switching what they are writing about and are mixing very different things.

Response: Thank you for your comments. We admit that it is not appropriate to measure “warmth” rather than “morality” in pilot experiment. In view of the weak logical relationship between the pilot experiment and the whole study, deleting the pilot experiment has no effect on the logic of the whole study and the integrity of the conclusions, we deleted the pilot experiment.

Second, the paper does not provide crucial information regarding the recruitment methods:

were there incentives given to participants?

did they complete informed consent?

what efforts were taken to ensure participant privacy and data protection?

what is the literature on the characteristics and reliability of Sojump respondents (e.g., compared to convenience samples of students or to representative samples)?

Response: Thank you for your comments. We added information about experimental remuneration and informed consent in the manuscript. The participants were recruited via the participant pool of the School of Psychology. The participants in the participant pool includes college students, graduate students and some social personnel. Participants who completed the experiment will receive 3 yuan for their participation. Before starting the experiment, the participants would read the informed consent form, and only with the consent will we collect the data of the participants. Since we did not record the specific information of the subjects, such as name and address, the participants were anonymous to us. Before the start of the experiment, we will also declare that we will not disclose the participants’ personal information, and all the data will only be used for scientific research and analysis. 

Sojump, an online platform similar to Mechanical Turk, is also known as Questionnaire Star or Wenjuanxing in Pinyin. To our knowledge, Sojump is currently the largest online data survey organization in China. Many researchers conduct questionnaires and behavioral experiments on it (Bai, B. Y., Liu, X. X., & Kou, Y., 2014; Klein, N., Grossmann, I., Uskul, A. K., Kraus, A. A., & Epley, N., 2015; Lien, C. H., & Cao, Y., 2014; Ruan, F., Fu, G., Zhou, M., Luo, L., Chen, J., Hua, W., at al., 2019; Yang, C., & Zhao, S., 2019; Zhang, J., Ziegler, M., & Paulhus, D. L., 2019). Since we recruited participants via the Psychology School’s participant pool, the respondents in our experiments were similar to convenience samples of students. Participants used the Sojump platform to complete the experiments on line. Moreover, the web surveys have the same effect as the print surveys (Huang, H., 2006). Therefore, we used this platform for data collection.

Third, the low numbers of respondents severely limit generalizability.

Response: Thank you for your comments. In the early version of the manuscript, the medium effect size is based on our unpublished manuscript on group potential preference. The effect size of that unpublished manuscript is similar to the effect size of potential preference in Tormala, Jia, & Norton’s (2012) study. In view of the fact that our study has not yet been published, we replaced the reference of the effect size of our unpublished manuscript with Tormala et al.’s (2012) study. In Tormala et al.’s study, the effect size is f=0.24. Under the conditions of α=.05 and a power of .90, the estimated sample size is the same as our actual sample size. 

Finally, the results of the study may in fact be caused by artifacts in the study design:

'moral improvement' was poorly operationalized, and respondents may have been more likely to give credence to reports that may be more salient (financial improvement and the potential for moral decline, as opposed to 'moral improvement' - do the authors know what that could consist of?)

Response: Thank you for your comments. We do often see reports of companies’ competence improvement and decline, as well as companies’ moral decline, in news and reports. We don’t often see news and reports on the companies’ moral improvement. We speculate that the lack of reports of the companies’ moral improvement may indicate that culturally, people may not attach importance to the companies’ moral improvement. 

We believe that the companies’ moral improvement does exist and people can understand the companies’ moral improvement, because in the manipulation check, participants could well distinguish between moral improvement and stable morality. Moreover, if people can use certain criteria to judge the company’s moral decline, then they should also be able to judge the company’s moral improvement by the same criteria. Therefore, referring to the evaluation criteria of corporate morality of Ethisphere Institute (see https://ethisphere.com/128-worlds-most-ethical-companies-for-2019/), we set up the company’s moral evaluation criteria as company ethics and compliance, company citizenship and responsibility, moral culture, company governance, and reputation. The decline in the company’s score in recent years indicates a decline in morality, while a rise in score indicates an improvement in morality.

However, we must admit that the hypothetical scenarios are not conducive to the generalization of research conclusions in reality. In the section of limitations and future directions, we discussed the impact of hypothetical scenarios on the generalization of research conclusions, as well as possible future research directions. 

Reviewer #4: In general, I believe this is an interesting project. The design seems adequate to test the hypotheses generated, though the methodology itself (particularly sample size) is of some concern. I have some concerns that I have outlined below:

Response: Thank you for your approval of research issue and research design. 

Please provide more information regarding the recruitment platform. How are participants selected? Are participants fairly compensated? Can you confirm that participants in one study did not also participate in another?

Response: Sojump is an online platform similar to Mechanical Turk. To our knowledge, Sojump is currently the largest online data survey organization in China. Many researchers conduct questionnaires and behavioral experiments on it (Bai, B. Y., Liu, X. X., & Kou, Y., 2014; Klein, N., Grossmann, I., Uskul, A. K., Kraus, A. A., & Epley, N., 2015; Lien, C. H., & Cao, Y., 2014; Ruan, F., Fu, G., Zhou, M., Luo, L., Chen, J., Hua, W., at al., 2019; Yang, C., & Zhao, S., 2019; Zhang, J., Ziegler, M., & Paulhus, D. L., 2019). Participants were recruited via the Psychology School’s participant pool. We published the experimental information through the WeChat platform of the participant pool. When people read the experimental recruitment information and the corresponding remuneration and want to attend the experiment, they would use computers or mobile devices to participate by linking or scanning QR codes. Participants would receive a compensation of 3 yuan by WeChat transfer. We prevent repeated participation of the same person by restricting access from the same IP address, and the current experiments are limited to adults older than 18 years old in mainland China. To prevent the same participants from participating in different experiments, we reminded people not to participate repeatedly if they have attended the former experiments in the advertisements, otherwise they would not be paid accordingly. We set the filtering system so that users with the same IP address cannot participate in different experiments. 

Given the reliance of this work on experimental methods throughout, I would have liked a bit more details about the actual experiments. Particularly, although I understand that the study materials are not in english, english translations of the actual stimulus materials would be very much appreciated.

Response: We translate the experimental materials as follows.

Experiment 1

Instruction 1 is the same in our four experiments, so we present Instruction 1 below, but not in the other three experiments’ materials to save space.

Instruction 1:

Welcome to our research. 

Center for Studies of Social Psychology, Central China Normal University supports the protection for human subjects involved in the study. The completion of this informed consent represents that you have reached the age of 18 and are willing to participate in the experiment. If you need to complain or have any other suggestions, please contact the researcher or the Human subjects Committee of Center for Studies of Social Psychology, CCNU.

Please check this item.

○I have read the consent form and voluntarily join this survey.

Instruction 2: 

We are interested in how people evaluate and make decisions based on information about groups (such as companies and organizations). In this study, we will present information about two different groups. Please read the information and answer the following questions.

Random: morality situation vs. competence situation

Morality situation: A corporate morality evaluation institution evaluates the companies’ moral status in various industries every year. The assessment includes company ethics and compliance, company citizenship and responsibility, moral culture, company governance, and reputation. 

Competence situation: A corporate competence evaluation institution evaluates the companies’ competence status in various industries every year. The assessment includes business performance, production and procurement ability, global marketing ability, international human resources, innovative research and development ability, and brand building ability.

The following descriptions in morality situation is very similar to that in the competence situation, so we combine the two to save space.

Company A and Company B are two companies with the same level of strength (morality) in the same industry.

The moral (competence) evaluation score of Company A has remained stable at about 95 in the past five years, and 95 in 2014 and 2018. The average score of moral (competence) evaluation of Company B is 89 in the past five years, 85 in 2014 and 93 in 2018, an average increase of about 2 points per year. The moral (competence) evaluation scores of Company A and Company B are shown in the following figure:

Morality (Competence) scores of Company A and Company B from 2014 to 2018.

*A morality (competence) score of 95 means that the morality (competence) ranking is in the top 5% of the industry, and the score of 85 means it is in the top 15%.

Questions:

If you were an applicant, to what extent would you be willing to join Company A?

○not at all ○2 ○3 ○4 ○5 ○6 ○very high

If you were an applicant, to what extent would you be willing to join Company B?

○not at all ○2 ○3 ○4 ○5 ○6 ○very high

If you were an applicant, which company would you prefer to join?

○Company A ○Company B

Which company do you think has higher moral (competence) achievements?

○Company A ○Company B

Which company do you think has higher potential for moral (competence) improvement?

○Company A ○Company B

Demographic information is collected at the end of each experiment, so we only present it here to save space.

Demographic information

What is your gender?

○Male ○Female 

What is your age?

( )

Experiment 2

Instruction 1: The same as Experiment 1.

Instruction 2: 

We are interested in how people evaluate and make decisions based on information about groups (such as companies and organizations). In this study, we will present information about two different groups. Please read the information and answer the following questions.

Random: morality situation vs. competence situation

Morality situation: A corporate morality evaluation institution evaluates the companies’ moral status in various industries every year. The assessment includes company ethics and compliance, company citizenship and responsibility, moral culture, company governance, and reputation. 

Competence situation: A corporate competence evaluation institution evaluates the companies’ competence status in various industries every year. The assessment includes business performance, production and procurement ability, global marketing ability, international human resources, innovative research and development ability, and brand building ability.

The following descriptions in morality situation is very similar to that in the competence situation, so we combine the two to save space.

Company A and Company B are two companies with the same level of strength (morality) in the same industry.

The moral (competence) evaluation score of Company A has remained stable at about 85 in the past five years, and 85 in 2014 and 2018. The average score of moral (competence) evaluation of Company B was 91 in the past five years, 95 in 2014 and 87 in 2018, an average decrease of about 2 points per year. The moral (competence) evaluation scores of Company A and Company B are shown in the following figure:

Morality (Competence) scores of Company A and Company B from 2014 to 2018.

*A morality (competence) score of 95 means that the morality (competence) ranking is in the top 5% of the industry, and the score of 85 means it is in the top 15%.

Questions:

If you were an applicant, to what extent would you be willing to join Company A?

○not at all ○2 ○3 ○4 ○5 ○6 ○very high

If you were an applicant, to what extent would you be willing to join Company B?

○not at all ○2 ○3 ○4 ○5 ○6 ○very high

If you were an applicant, which company would you prefer to join?

○Company A ○Company B

Which company do you think has higher moral (competence) achievements?

○Company A ○Company B

Which company do you think has higher potential for moral (competence) decline?

○Company A ○Company B

Demographic information：the same as Experiment 1

Experiment 3

Instruction 1: The same as Experiment 1.

Instruction 2: 

We are interested in how people evaluate and make decisions based on information about groups (such as companies and organizations). In this study, we will present information about three different groups. Please read the information and answer the following questions.

Random: potential for improvement vs. stable performance vs. potential for decline

A corporate morality evaluation institution evaluates the companies’ moral status in various industries every year. The assessment includes company ethics and compliance, company citizenship and responsibility, moral culture, company governance, and reputation.

Company A, Company B and Company C are three companies with the same level of strength in the same industry.

Potential for improvement: The moral evaluation scores of Company A were 92.9 in 2016 and 95 in 2018. The average score of moral evaluation of Company A was 94 in the past three years. 

Stable performance: The moral evaluation scores of Company B were 94.9 in 2016 and 95 in 2018. The average score of moral evaluation of Company B was 95 in the past three years. 

Potential for decline: The moral evaluation scores of Company C were 97.1 in 2016 and 95 in 2018. The average score of moral evaluation of Company C was 96 in the past three years.

The moral evaluation scores of Company A, Company B and Company C are shown in the following figure:

Morality scores of Company A, Company B, and Company C from 2016 to 2018.

*A morality score of 95 means that the morality ranking is in the top 5% of the industry, and the score of 96 means it is in the top 4%.

Questions:

If you were an applicant, to what extent would you be willing to join Company A?

○not at all ○2 ○3 ○4 ○5 ○6 ○very high

If you were an applicant, to what extent would you be willing to join Company B?

○not at all ○2 ○3 ○4 ○5 ○6 ○very high

If you were an applicant, to what extent would you be willing to join Company C?

○not at all ○2 ○3 ○4 ○5 ○6 ○very high

If you were an applicant, which company would you prefer to join?

○Company A ○Company B ○Company C

Please rank the companies’ potential for moral improvement of Company A, Company B, and Company C, and rank the one you think has the highest potential as the first and the one with the lowest potential as the third. 

○Company A ○Company B ○Company C

Please rank the companies’ potential for moral decline of Company A, Company B, and Company C, and rank the one you think has the highest potential as the first and the one with the lowest potential as the third. 

○Company A ○Company B ○Company C

Demographic information：the same as Experiment 1

Experiment 4

Instruction 1: The same as Experiment 1.

Instruction 2: 

We are interested in how people evaluate and make decisions based on information about groups (such as companies and organizations). In this study, we will present information about a group. Please read the information and answer the following questions.

Random: stable performance vs. potential for improvement vs. potential for decline

A corporate morality evaluation institution evaluates the companies’ moral status in various industries every year. The assessment includes company ethics and compliance, company citizenship and responsibility, moral culture, company governance, and reputation.

Stable performance: The moral evaluation scores of Company A were 95 in 2016 and 2018. The average score of moral evaluation of Company A was 95 in the past three years.

Potential for improvement: The moral evaluation scores of Company A were 94 in 2016 and 96 in 2018. The average score of moral evaluation of Company A was 95 in the past three years. 

Potential for decline: The moral evaluation scores of Company A were 96 in 2016 and 94 in 2018. The average score of moral evaluation of Company A was 95 in the past three years.

The moral evaluation scores of Company A are shown in the following figure:

Morality scores of Company A from 2016 to 2018.

*A morality score of 95 means that the morality ranking is in the top 5% of the industry, and the score of 96 means it is in the top 4%.

Questions:

If you were an applicant, to what extent would you be willing to join Company A?

○not at all ○2 ○3 ○4 ○5 ○6 ○very high

What do you think is Company A’s potential for moral improvement?

○very low ○2 ○3 ○4 ○5 ○6 ○very high

Demographic information：the same as Experiment 1

Throughout, the sample sizes are quite small. Although power analyses are reported in one part of the manuscript, they are not reported for all experiments. The size of samples leaves me concerned about the generalizability of the findings.

Response: Thank you for your comments. We adjusted the parameters to estimate the sample size of experiment 1. In the early version of manuscript, the medium effect size is based on our unpublished manuscript on group potential preference. The effect size of that unpublished manuscript is similar to the effect size of potential preference in Tormala, Jia, & Norton’s (2012) study. In view of the fact that our study has not yet been published, we replaced the reference of the effect size of our unpublished manuscript with Tormala et al.’s (2012) study. In Tormala et al.’s study, the effect size is f=0.24. Under the conditions of α=.05 and a power of .90, the estimated sample size is the same as our actual sample size. 

Experiment 1:

Do not use terms like: "marginally significant." This is a meaningless phrase that relies on a statistic being close to an arbitrary cutoff. If your a-priori power analyses indicated that your alpha level was .05, anything that does not meet this is not significant. Please delete this language throughout.

Response: Thank you for your suggestion. We accept your suggestion and deleted the term of “marginally” in the manuscript. 

For Experiment 2, how was sample size determined?

For Experiment 3, how was sample size determined?

For Experiment 4, how was sample size determined?

In each of the above examples, is it assumed that the same power analyses from Experiment 1 still applied?

Response: Thank you for your comments. We think that the power analysis of Experiment 1 is not suitable for the other three experiments. Therefore, we conducted a sensitivity power analysis in experiment 2, 3 and 4 respectively. The results showed that our sample sizes were sufficient to detect a medium effect size. Although our sample sizes may not be very large, we think that the sample sizes of the experiments are sufficient to detect a medium effect size.

References

Bai, B. Y., Liu, X. X., & Kou, Y. (2014). Belief in a just world lowers perceived intention of corruption: the mediating role of perceived punishment. PLoS One, 9(5), e97075. doi:10.1371/journal.pone.0097075

Huang, H. (2006). Do print and Web surveys provide the same results? Computers in Human Behavior, 22(3), 334-350. doi:10.1016/j.chb.2004.09.012

Klein, N., Grossmann, I., Uskul, A. K., Kraus, A. A., & Epley, N. (2015). It pays to be nice, but not really nice: Asymmetric reputations from prosociality across 7 countries. Judgment & Decision Making, 10(4), 355-364.

Lien, C. H., & Cao, Y. (2014). Examining WeChat users’ motivations, trust, attitudes, and positive word-of-mouth: Evidence from China. Computers in Human Behavior, 41, 104-111. doi:10.1016/j.chb.2014.08.013

Ruan, F., Fu, G., Zhou, M., Luo, L., Chen, J., Hua, W., . . . Wang, J. (2019). Application of the Chinese version of Zelaya’s HIV-related stigma scale to undergraduates in mainland China. BMC Public Health, 19(1), 1708. doi:10.1186/s12889-019-8054-9

Tormala, Z. L., Jia, J. S., & Norton, M. I. (2012). The preference for potential. Journal of Personality and Social Psychology, 103(4), 567-583. doi:10.1037/a0029227

Yang, C., & Zhao, S. (2019). Determining preference for potential: The role of perceived economic mobility. Social Behavior and Personality: an international journal, 47(6), e8019. doi:10.2224/sbp.8019

Zhang, J., Ziegler, M., & Paulhus, D. L. (2019). Development and evaluation of the short Dark Triad – Chinese version (SD3-C). Current Psychology. doi:10.1007/s12144-019-00272-3

Attachment Submitted filename: Response_to_Reviewers.docx Click here for additional data file.

# Peer review: Decision Letter 1

**DOI:** 10.1371/journal.pone.0236748.r003

**Authors:** Baogui Xin

17 Jun 2020

PONE-D-20-05265R1

The preference for potential in competence, not in morality: Asymmetric biases regarding a group's potential for moral improvement and decline

PLOS ONE

Dear Dr. Zuo,

Thank you for submitting your manuscript to PLOS ONE. After careful consideration, we feel that it has merit but does not fully meet PLOS ONE’s publication criteria as it currently stands. Therefore, we invite you to submit a revised version of the manuscript that addresses the points raised during the review process.

I recommend that it should be revised taking into account the changes requested by Reviewers. I would like to give you the last chance to revise your manuscript. To speed the review process, the manuscript will only be reviewed by the Academic Editor in the next round.

Please submit your revised manuscript by Aug 01 2020 11:59PM. If you will need more time than this to complete your revisions, please reply to this message or contact the journal office at plosone@plos.org. When you're ready to submit your revision, log on to https://www.editorialmanager.com/pone/ and select the 'Submissions Needing Revision' folder to locate your manuscript file.

Please include the following items when submitting your revised manuscript:

- A rebuttal letter that responds to each point raised by the academic editor and reviewer(s). You should upload this letter as a separate file labeled 'Response to Reviewers'.
- A marked-up copy of your manuscript that highlights changes made to the original version. You should upload this as a separate file labeled 'Revised Manuscript with Track Changes'.
- An unmarked version of your revised paper without tracked changes. You should upload this as a separate file labeled 'Manuscript'.

If you would like to make changes to your financial disclosure, please include your updated statement in your cover letter. Guidelines for resubmitting your figure files are available below the reviewer comments at the end of this letter.

If applicable, we recommend that you deposit your laboratory protocols in protocols.io to enhance the reproducibility of your results. Protocols.io assigns your protocol its own identifier (DOI) so that it can be cited independently in the future. For instructions see: http://journals.plos.org/plosone/s/submission-guidelines#loc-laboratory-protocols

We look forward to receiving your revised manuscript.

Kind regards,

Baogui Xin, Ph.D.

Academic Editor

PLOS ONE

[Note: HTML markup is below. Please do not edit.]

Reviewers' comments:

Reviewer's Responses to Questions

**Comments to the Author**

1. If the authors have adequately addressed your comments raised in a previous round of review and you feel that this manuscript is now acceptable for publication, you may indicate that here to bypass the “Comments to the Author” section, enter your conflict of interest statement in the “Confidential to Editor” section, and submit your "Accept" recommendation.

Reviewer #1: All comments have been addressed

Reviewer #2: All comments have been addressed

**********

2. Is the manuscript technically sound, and do the data support the conclusions?

The manuscript must describe a technically sound piece of scientific research with data that supports the conclusions. Experiments must have been conducted rigorously, with appropriate controls, replication, and sample sizes. The conclusions must be drawn appropriately based on the data presented. 

Reviewer #1: Yes

Reviewer #2: Yes

**********

3. Has the statistical analysis been performed appropriately and rigorously? 

Reviewer #1: Yes

Reviewer #2: Yes

**********

4. Have the authors made all data underlying the findings in their manuscript fully available?

The PLOS Data policy requires authors to make all data underlying the findings described in their manuscript fully available without restriction, with rare exception (please refer to the Data Availability Statement in the manuscript PDF file). The data should be provided as part of the manuscript or its supporting information, or deposited to a public repository. For example, in addition to summary statistics, the data points behind means, medians and variance measures should be available. If there are restrictions on publicly sharing data—e.g. participant privacy or use of data from a third party—those must be specified.

Reviewer #1: Yes

Reviewer #2: Yes

**********

5. Is the manuscript presented in an intelligible fashion and written in standard English?

PLOS ONE does not copyedit accepted manuscripts, so the language in submitted articles must be clear, correct, and unambiguous. Any typographical or grammatical errors should be corrected at revision, so please note any specific errors here.

Reviewer #1: Yes

Reviewer #2: Yes

**********

6. Review Comments to the Author

Please use the space provided to explain your answers to the questions above. You may also include additional comments for the author, including concerns about dual publication, research ethics, or publication ethics. (Please upload your review as an attachment if it exceeds 20,000 characters)

Reviewer #1: Revision: PONE-D-20-05265

The manuscript has improved considerably compared to the initial submission. It is good to see that the authors incorporated the literature suggested in the previous review rounds.

However, I have several more comments on this manuscript:

In experiment 2, the morality condition does not show significant results, p = .12, but is interpreted as if significant. This interpretation needs to be consistent across the paper, as insignificant results cannot be interpreted the same way as significant ones. The same problem appears in experiment 3 in the interpretation of the manipulation check, where p=.06 is interpreted as significant.

In all experiments, it’s unclear what the authors mean when they say that the same came from a psychology subject pool and was then conducted on an online platform similar to MTurk. Are all participants students who simply use the platform to receive their participation reward? If so, the analogy to MTurk is confusing, as people on MTurk are different in terms of demographics from students, and students would not be asked to use MTurk to complete studies.

On page 21, the first sentence of the results section is confusing. A significant effect of what? Of the experimental condition I assume, but this needs to be explicit.

The term asymmetry is not entirely clear, as it refers to the different effect of improvement vs. decline. I think this would be more clearly named non-linearity, or at the very least the asymmetry should be very clearly defined.

In the Discussion, I’m confused by the authors’ interpretation (p.24) that: “It seems that the morality of for-profit companies should neither improve nor decline. That situation creates a moral dilemma for for-profit companies.” Improvement on morality certainly won’t hurt, as their studies show, so why should a company not improve? Do they mean that given resource constraints, it might not be wise to invest in improvement?

More discussion of potential moderators of the asymmetry would be helpful. Would company size, entitativity, homogeneity influence the pattern? Would the effect differ depending on whether the morality is described as top-down actions from the company’s leadership versus part of a general company culture?

Reviewer #2: One last comment: Regarding the sentence "the improvement and decline of morality may correspond to different dimensions, such as prescriptive and proscriptive morality, respectively", I found it reluctant to categorize moral improvement/decline into prescriptive/proscriptive morality as "two moral dimensions". Dimensions are usually descriptive of different contents but not the trend of the same content. I listed this reference earlier, but may be misinterpreted by the authors. A better way to put it may be the "bad is stronger than good" effect. The authors may think how to better structure the relevance of the theories and their findings.

Baumeister, R. F., Bratslavsky, E., Finkenauer, C., & Vohs, K. D. (2001). Bad is stronger than good. Review of General Psychology, 5(4), 323-370.

**********

7. PLOS authors have the option to publish the peer review history of their article (what does this mean?). If published, this will include your full peer review and any attached files.

If you choose “no”, your identity will remain anonymous but your review may still be made public.

**Do you want your identity to be public for this peer review?** For information about this choice, including consent withdrawal, please see our Privacy Policy.

Reviewer #1: No

Reviewer #2: No

[NOTE: If reviewer comments were submitted as an attachment file, they will be attached to this email and accessible via the submission site. Please log into your account, locate the manuscript record, and check for the action link "View Attachments". If this link does not appear, there are no attachment files.]

While revising your submission, please upload your figure files to the Preflight Analysis and Conversion Engine (PACE) digital diagnostic tool, https://pacev2.apexcovantage.com/. PACE helps ensure that figures meet PLOS requirements. To use PACE, you must first register as a user. Registration is free. Then, login and navigate to the UPLOAD tab, where you will find detailed instructions on how to use the tool. If you encounter any issues or have any questions when using PACE, please email PLOS at figures@plos.org. Please note that Supporting Information files do not need this step.

Attachment Submitted filename: Revision_20_05265.docx Click here for additional data file.

# Author comment: Author response to Decision Letter 1

**DOI:** 10.1371/journal.pone.0236748.r004

3 Jul 2020

Dear Editor and Reviewers,

Thank you for your letter. We appreciate the reviewers’ approval of the revised contents and thank you for your new comments concerning our revised manuscript entitled “The preference for potential in competence, not in morality: Asymmetric biases regarding a group’s potential for moral improvement and decline” (ID: PONE-D-20-05265R1). These new comments are of great value to the improvement of our manuscript. We studied the comments carefully and have made corrections, hoping to meet your approval. The main corrections in the manuscript and the responds to the reviewers’ comments are as following:

Responds to the reviewers’ comments

Reviewer #1: Revision: PONE-D-20-05265

The manuscript has improved considerably compared to the initial submission. It is good to see that the authors incorporated the literature suggested in the previous review rounds.

Response: Thank you for your approval of the revised manuscript.

However, I have several more comments on this manuscript:

In experiment 2, the morality condition does not show significant results, p = .12, but is interpreted as if significant. This interpretation needs to be consistent across the paper, as insignificant results cannot be interpreted the same way as significant ones. The same problem appears in experiment 3 in the interpretation of the manipulation check, where p=.06 is interpreted as significant. 

Response: Thank you for your comments. Although the result of the individual choice in the morality condition in experiment 2 and one of the results of the manipulation check in experiment 3 were consistent with our expectations, they did not reach statistical significance. To maintain the consistent of the interpretation of statistical significance, we revised the description of those two results. The corresponding changes in our manuscript are as follows.

Similarly, in the morality condition, there was a tendency for more participants to select to join the stably moderate-performance company (30/49) than the high-performance company with high potential for decline (19/49; χ2(1) = 2.47, p = .12).

(please see changes on page 15)

Wilcoxon signed-rank tests showed that Company A (1.58) was ranked higher than Company B (2.07) (Z = 3.12, p < .01) and Company C (2.35) (Z = 3.02, p < .01), and Company B was ranked higher than Company C (Z = 1.89, p = .06), although this result did not reach statistical significance.

(please see changes on page 17)

In all experiments, it’s unclear what the authors mean when they say that the sample came from a psychology subject pool and was then conducted on an online platform similar to MTurk. Are all participants students who simply use the platform to receive their participation reward? If so, the analogy to MTurk is confusing, as people on MTurk are different in terms of demographics from students, and students would not be asked to use MTurk to complete studies. 

Response: Thank you for your comments. Our participants came from the Psychology School’s participant pool. We posted the recruitment information on the participants pool’s online platform, and the participants who were interested in the experiment participated the experiment through Sojump. Because there is a cooperative relationship between Sojump and WeChat, the participants who finished the experiments on Sojump were paid through WeChat transfer. If there are no college students in MTurk, then Mturk and Sojump are indeed different. Therefore, we delete that analogical statement from our manuscript. The corresponding changes in our manuscript are as follows.

Because web surveys are as effective as print surveys [40] and save more resources, we chose to use the online survey platform Sojump (https://www.sojump.com). A total of 92 Chinese adults (19 men and 73 women; Mage = 20.40 years, SD = 2.46) completed the experiment individually online.

(please see changes on page 9)

On page 21, the first sentence of the results section is confusing. A significant effect of what? Of the experimental condition I assume, but this needs to be explicit.

Response: Thank you for your comments. We changed the statement of this result to make it clearer. The corresponding changes in our manuscript are as follows.

An ANOVA revealed a significant effect of characteristic (F(2, 147) = 53.67, p < .001, η2 p = .42).

(please see changes on page 21)

The term asymmetry is not entirely clear, as it refers to the different effect of improvement vs. decline. I think this would be more clearly named non-linearity, or at the very least the asymmetry should be very clearly defined.

Response: Thank you for your comments. To convey the meaning of the asymmetric biases more clearly, we defined this concept explicitly in the manuscript. The corresponding changes in our manuscript are as follows.

Specifically, the asymmetric biases in this article are defined as a non-preference for the potential for moral improvement, but an aversion to the potential for moral decline.

(please see changes on page 5-6)

In the Discussion, I’m confused by the authors’ interpretation (p.24) that: “It seems that the morality of for-profit companies should neither improve nor decline. That situation creates a moral dilemma for for-profit companies.” Improvement on morality certainly won’t hurt, as their studies show, so why should a company not improve? Do they mean that given resource constraints, it might not be wise to invest in improvement? 

Response: Thank you for your comments. The statement of “the morality of for-profit companies should neither improve nor decline” is not very clear. What we are trying to convey is that the moral improvement of for-profit companies may lead to a decline in people’s evaluation of their competence, so for-profit companies’ moral improvement may have negative effects. To make it clearer, we changed the corresponding contents. The corresponding changes in our manuscript are as follows.

The situation is a little more subtle for the morality of for-profit companies. Although for-profit companies have made important contributions to social progress, they are stereotypically depicted as immoral and harmful to society [44]. On the contrary, the moral improvement of for-profit companies is considered to hinder their competence [34]. It seems that for-profit companies should pay more attention to the development of their competence. However, no matter how competitive a for-profit company is, any moral decline or scandal may put the company in jeopardy [36-38]. To sum up, the moral improvement of for-profit companies may lead to a decline in people’s evaluation of the companies’ competence, while the companies’ moral decline may lead to people’s worse overall impressions of them. It seems that both the moral improvement and decline of for-profit companies may have negative effects. That situation creates a moral dilemma for for-profit companies. Future research could further test the effect of for-profit corporate moral dilemma.

(please see changes on page 24)

More discussion of potential moderators of the asymmetry would be helpful. Would company size, entitativity, homogeneity influence the pattern? Would the effect differ depending on whether the morality is described as top-down actions from the company’s leadership versus part of a general company culture? 

Response: Thank you for your insightful suggestions. According to your suggestion in the previous review, we discussed the influence of group size on the asymmetric biases in the previous revision. Following your suggestion in the current review, we found that the entitativity could also affect people’s evaluation of group morality. Therefore, we discussed why the entitativity might also be a potential moderator of the asymmetry. Moreover, the characteristics of group morality and the individual motivation may also have an impact on the asymmetry. We discussed those issues in the manuscript. The corresponding changes in our manuscript are as follows.

For example, the size and the entitativity of a group can affect people’s moral judgment of the group. Small groups are perceived to be more communal and trustworthy than big ones [51, 52]. Therefore, the moral potential of small companies may have a greater impact than big companies on an applicant’s willingness to join. Perceived entitativity could make people’s moral judgment of the group more extreme. People are more averse to the immoral behaviors of the high entitativity group than the low entitativity group, but they prefer the moral behaviors of the high entitativity group [53, 54]. Therefore, group entitativity may moderate people’s attitude towards the group’s moral improvement and decline. In addition, future research can explore the moderating effect of the characteristics of a company’s morality and the individual motivation on the asymmetric biases regarding a group’s potential for moral improvement and decline. The company’s moral improvement and decline may be caused by the corporate culture or the top-down actions of the company’s leadership. Because the corporate culture is relatively stable, people may deem that a company’s moral improvement and decline driven by leadership is not as lasting as the changes brought about by the corporate culture. Therefore, the corporate culture may have a greater impact on people’s attitude towards a company’s moral improvement and decline than the company’s leadership does. Moreover, applicants who want to change the future of the company may value the company’s moral potential more than those who passively accept the status quo. Overall, testing the boundary conditions of asymmetric biases regarding a group’s moral potential would facilitate more practical application of study findings.

(please see changes on page 25-26)

Reviewer #2: One last comment: Regarding the sentence "the improvement and decline of morality may correspond to different dimensions, such as prescriptive and proscriptive morality, respectively", I found it reluctant to categorize moral improvement/decline into prescriptive/proscriptive morality as "two moral dimensions". Dimensions are usually descriptive of different contents but not the trend of the same content. I listed this reference earlier, but may be misinterpreted by the authors. A better way to put it may be the "bad is stronger than good" effect. The authors may think how to better structure the relevance of the theories and their findings.

Baumeister, R. F., Bratslavsky, E., Finkenauer, C., & Vohs, K. D. (2001). Bad is stronger than good. Review of General Psychology, 5(4), 323-370. 

Response: Thank you for your comments. It is indeed inappropriate to directly associate moral improvement and decline with prescriptive and proscriptive morality, respectively. We proposed that people might associate moral improvement and decline with moral and immoral behaviors, respectively. It may be that people’s asymmetric attitudes towards the moral and immoral behaviors lead to people’s asymmetric biased regarding the moral potential. However, we think that the “bad is stronger than good” effect may also have an impact on the asymmetric biases regarding the moral potential. The corresponding changes in our manuscript are as follows.

However, the asymmetric biases regarding the potential for moral improvement and decline indicate that people may process the information of moral improvement and decline differently. Although we manipulated the potential for moral improvement and decline in a similar manner, people may have associated moral improvement and decline with moral and immoral behaviors, respectively. Research shows that people do not force others to become more moral, but they cannot tolerate others becoming more immoral [29]. Consequently, people who perform different degrees of good deeds may be perceived to have the same degree of kindness, but the more bad things they do, the worse they are [31, 32]. In addition, bad actions are often more powerful than good ones [43]. Therefore, although people have no preference for the potential for moral improvement, they are averse to the potential for moral decline.

(please see changes on page 23)

Attachment Submitted filename: Response_to_Reviewers.docx Click here for additional data file.

# Editor report: Decision Letter 2

**DOI:** 10.1371/journal.pone.0236748.r005

**Authors:** Baogui Xin

14 Jul 2020

The preference for potential in competence, not in morality: Asymmetric biases regarding a group's potential for moral improvement and decline

PONE-D-20-05265R2

Dear Dr. Zuo,

We’re pleased to inform you that your manuscript has been judged scientifically suitable for publication and will be formally accepted for publication once it meets all outstanding technical requirements.

Within one week, you’ll receive an e-mail detailing the required amendments. When these have been addressed, you’ll receive a formal acceptance letter and your manuscript will be scheduled for publication.

An invoice for payment will follow shortly after the formal acceptance. To ensure an efficient process, please log into Editorial Manager at http://www.editorialmanager.com/pone/, click the 'Update My Information' link at the top of the page, and double check that your user information is up-to-date. If you have any billing related questions, please contact our Author Billing department directly at authorbilling@plos.org.

If your institution or institutions have a press office, please notify them about your upcoming paper to help maximize its impact. If they’ll be preparing press materials, please inform our press team as soon as possible -- no later than 48 hours after receiving the formal acceptance. Your manuscript will remain under strict press embargo until 2 pm Eastern Time on the date of publication. For more information, please contact onepress@plos.org.

Kind regards,

Baogui Xin, Ph.D.

Academic Editor

PLOS ONE

Additional Editor Comments (optional):

Reviewers' comments:

# Editor report: Acceptance letter

**DOI:** 10.1371/journal.pone.0236748.r006

**Authors:** Baogui Xin

16 Jul 2020

PONE-D-20-05265R2 

The preference for potential in competence, not in morality: Asymmetric biases regarding a group's potential for moral improvement and decline 

Dear Dr. Zuo:

I'm pleased to inform you that your manuscript has been deemed suitable for publication in PLOS ONE. Congratulations! Your manuscript is now with our production department. 

If your institution or institutions have a press office, please let them know about your upcoming paper now to help maximize its impact. If they'll be preparing press materials, please inform our press team within the next 48 hours. Your manuscript will remain under strict press embargo until 2 pm Eastern Time on the date of publication. For more information please contact onepress@plos.org.

If we can help with anything else, please email us at plosone@plos.org. 

Thank you for submitting your work to PLOS ONE and supporting open access. 

Kind regards, 

PLOS ONE Editorial Office Staff

on behalf of

Professor Baogui Xin 

Academic Editor

PLOS ONE
